# Natural drugs modulate MAPK: targets and strategies for liver fibrosis treatment

**DOI:** 10.3389/fphar.2026.1792301

**Published:** 2026-03-19

**Authors:** Qingxin Luan, Jianbo Wang, Wei Kong, Song Guo

**Affiliations:** Affiliated Hospital of Shandong University of Traditional Chinese Medicine, Jinan, China

**Keywords:** hepatic stellate cells, liver fibrosis, MAPK, matrix deposition, natural drugs

## Abstract

Liver fibrosis (LF) as a critical pathological stage in the progression of various chronic liver diseases toward cirrhosis and hepatocellular carcinoma, has become a severe global public health challenge posing a serious threat to patient health. Consequently, there is an urgent need to develop novel, highly effective therapeutic drugs and intervention strategies. The application of traditional Chinese medicine (TCM) formulas and natural drugs leveraging their unique advantages of multi-target and multi-pathway mechanisms, is increasingly attracting attention in the field of LF treatment. In recent years, a growing number of studies have focused on the therapeutic value of natural drugs and TCM formulas in treating LF. This review systematically explores the clinical application potential and core therapeutic mechanisms of natural drugs and TCM formulas in LF treatment, covering natural compounds such as flavonoids, terpenoids, and glycosides, as well as TCM formulas like Tianhuang Formula and Yiqu Ronggan Formula. We demonstrate that multiple key bioactive components, such as quercetin, curcumin, and astragaloside IV, exhibit beneficial effects in LF intervention. These effects primarily occur through modulating critical pathogenic drivers including hepatic stellate cell (HSC) activation, inflammatory responses, and oxidative stress. The elucidated therapeutic mechanisms primarily include inhibiting HSC activation and proliferation, regulating inflammatory pathways, alleviating oxidative stress damage, reducing excessive extracellular matrix (ECM) deposition, and restoring liver tissue microenvironment homeostasis. This preclinical evidence supports the potential efficacy of natural drugs and TCM formulas as complementary and alternative treatment options for LF. However, most existing evidence is based on *in vitro* cell experiments and animal model studies, significantly limiting its clinical translational value. Further rigorous, multicenter, large-scale clinical trials are needed to validate the efficacy and long-term safety of natural drugs and TCM formulas in patients with LF, thereby providing robust evidence-based medical support for their clinical application.

## 1 Introduction

Liver fibrosis (LF) represents a progressive pathological remodeling process induced by chronic liver injury. Its core pathological features include abnormal activation and proliferation of hepatic stellate cells (HSCs) alongside excessive extracellular matrix (ECM) deposition. Without timely intervention, the disease can progressively advance to cirrhosis and even deteriorate into hepatocellular carcinoma, posing a severe threat to patient health and life (Yang J. et al., 2023). As a progressive and complex disease, LF not only significantly diminishes patients’ quality of life and worsens clinical outcomes but has also emerged as a major global public health concern. According to World Health Organization (WHO) statistics, over 100 million people worldwide suffer from cirrhosis, posing a formidable challenge to global healthcare systems (Zamani et al., 2025). The clinical manifestations of LF exhibit pronounced disease-stage dependence: symptoms are subtle and difficult to detect in the early stages. Upon progression, typical symptoms such as fatigue, hepatic dull pain, jaundice, and ascites emerge successively. By this stage, irreversible liver damage has already occurred, leading to a sharp decline in quality of life (Weissenborn, 2019). Notably, the reversibility of LF progressively diminishes with disease progression, making early, precise intervention crucial to halt its progression to end-stage liver disease. However, this condition involves a complex pathophysiological regulatory network involving multiple cell types and signaling pathways, presenting significant challenges for clinical diagnosis and therapeutic strategy development that demand urgent breakthroughs.

The pathogenesis of LF encompasses multiple pathways, including hepatocyte injury, immune cell infiltration, HSCs activation, and ECM metabolic imbalance. Key drivers include genetic susceptibility, immune microenvironment dysregulation, metabolic abnormalities, and environmental exposures (Khanam et al., 2021). Current clinical treatment strategies primarily focus on etiological control, symptomatic support, and anti-fibrotic interventions. Commonly used drugs include antiviral agents, antioxidants, and immunomodulators. While these can delay disease progression to some extent, they lack specific targeted anti-fibrotic drugs. Long-term use is also associated with side effects such as drug resistance and hepatic/renal dysfunction (Pan et al., 2024). Therefore, identifying novel anti-fibrotic targets and therapeutic strategies with high specificity and safety has become an urgent need in LF research. Multiple studies confirm that the MAPK pathway plays a central regulatory role in the pathological process of LF, with its abnormal activation serving as a key molecular mechanism driving HSCs activation, promoting inflammatory responses, and facilitating ECM deposition (Fang et al., 2023). Within the fibrotic liver microenvironment, sustained MAPK pathway activation exacerbates inflammatory injury and fibrotic remodeling by promoting HSC-to-myofibroblast transdifferentiation and inducing proinflammatory cytokines and fibrosis-related genes (e.g., α-SMA, COL1A1), highlighting its significant potential as an anti-fibrotic therapeutic target.

As a vital source for new drug development, natural drugs have garnered significant attention in antifibrotic drug research due to their structural diversity, broad biological activities, and relatively low toxicity. Numerous modern drugs originate from natural drugs and their derivatives. For example, curcumin extracted from medicinal plants has been demonstrated to possess significant hepatoprotective and antifibrotic activities. Compared to traditional single-target drugs, natural drugs often exert pharmacological effects through synergistic mechanisms involving “multiple targets and signaling pathways,” exhibiting unique therapeutic advantages when intervening in complex diseases such as LF. However, in the existing literature, although recent reviews by scholars such as (Liang et al., 2025; Cui et al., 2025; Zhou et al., 2024) have explored the holistic intervention effects of TCM and natural active ingredients on various fibrotic diseases, these studies are largely confined to broad overviews at the macro level. These studies not only fail to deeply focus on the core driving role of the MAPK pathway in the pathological progression of LF but also lack a systematic explanation of how natural medicines precisely target this pathway to reverse the mechanism of LF. Given this, this review aims to systematically dissect the molecular regulatory mechanisms of the MAPK pathway in the onset and progression of LF, with a particular emphasis on elucidating the pharmacological basis by which natural drugs exert anti-fibrotic effects through specific targeting of this pathway. Furthermore, this paper will explore the application prospects and challenges of natural drugs in the targeted treatment of LF, aiming to provide a solid theoretical basis and innovative insights for the subsequent development and clinical translation of new anti-fibrotic drugs.

## 2 Methods and literature search strategy

**FIGURE 1:**
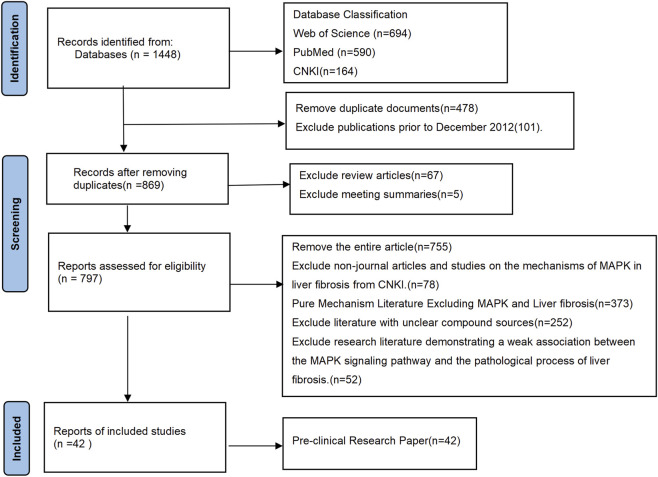
Flowchart of literature search.

This study systematically searched the China National Knowledge Infrastructure (CNKI), PubMed, and Web of Science databases. The literature collection period was set from December 2012 to September 2025. Literature retrieval was conducted according to the PRISMA checklist (https://www.prisma-statement.org). The search strategy combined free-text keywords with Medical Subject Headings (MeSH). Keywords primarily focused on the intersection of intervention measures including “natural compounds,” “traditional Chinese medicine formulas” or “Chinese herbal extracts” and the target disease “liver fibrosis.” Included studies met all of the following criteria: (1) Experiments were conducted using *in vivo* (animal) or *in vitro* (cell models); (2) The drugs used were extracts with clearly defined chemical compositions or identified Chinese herbal monomers/metabolites; (3) Demonstrated regulatory effects on MAPK pathway, manifested as reduced inflammatory injury, inhibition of abnormal angiogenesis, improved oxidative stress (OS) status, suppression of HSC activation, or decreased abnormal accumulation of ECM. Conversely, studies meeting the following criteria were excluded: First, 478 duplicated publications were excluded; Second, outdated studies published before December 2012 (101 articles), review articles (67 articles), and conference abstracts (5 articles) were removed to avoid interference from non-original research data; Third, non-journal articles and studies from CNKI focusing solely on the mechanism of MAPK pathway in LF (78 articles) were excluded, as were purely mechanistic literature addressing only MAPK pathway or LF (373 articles). Fourth, studies with unclear compound sources (252 articles) and those demonstrating weak associations between the MAPK pathway and the pathological process of LF (52 articles) were excluded. Ultimately, 42 studies met the inclusion criteria and were selected for in-depth discussion in this review (Figure 1).

## 3 Overview of the MAPK pathway

The mitogen-activated protein kinase (MAPK) pathway represents a highly conserved core signal transduction system in eukaryotic evolution. Its defining feature is a three-tiered enzyme-linked phosphorylation cascade (“MAPKKK-MAPKK-MAPK”) that establishes a precise pathway for transducing extracellular signals to the nucleus (Avkiran and Marber, 2010). This pathway family comprises key subtypes such as extracellular signal-regulated kinase (ERK), p38 MAPK, and c-Jun N-terminal kinase (JNK). These subtypes form complex signaling regulatory networks through synergistic interactions, which can specifically recognize and integrate diverse extracellular signals including physiological regulatory signals such as growth factors, pro-inflammatory cytokines, and hormones, as well as environmental stress signals like OS and osmotic fluctuations. Through cascade reactions, these signals are progressively amplified and transmitted to the cell nucleus, ultimately achieving efficient conversion of extracellular signals into nuclear gene expression regulatory commands. This mechanism serves as a critical hub for cells to perceive external environments and regulate internal physiological activities (Hepworth and Hinton, 2021).

As a core molecular pathway governing cellular fate, MAPK profoundly engages in fundamental cellular processes such as gene transcription regulation, protein post-translational modification, and cytoskeletal remodeling. Consequently, it exerts precise control over critical biological behaviors including cell proliferation, differentiation, migration, and apoptosis (Yue and López, 2020). Within the regulation of inflammatory immune responses, MAPK occupies a central position: by controlling the activation, proliferation, and differentiation states of immune cells, mediating the balance of pro-inflammatory/anti-inflammatory cytokine secretion, and regulating the synthesis and release of inflammatory mediators, it serves as a pivotal node connecting upstream stress signals to downstream inflammatory responses. It is a core regulator in the initiation of acute inflammation and the maintenance of chronic inflammation (Arthur and Ley, 2013).

**FIGURE 2:**
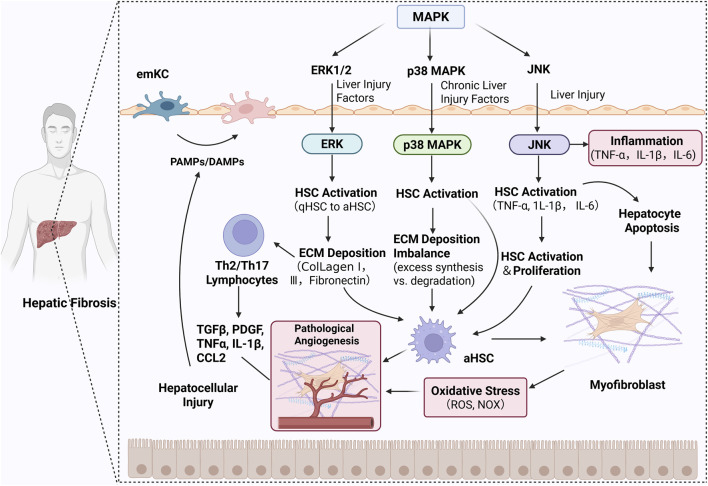
statetic diagram of MAPK involvement in LF mechanisms.

Notably, abnormal activation of MAPK exhibits tight molecular associations with the development of LF. Its core regulatory mechanism directly targets two critical pathological nodes in the fibrosis process: activation of HSCs and excessive deposition of ECM (Jin et al., 2022). When liver tissue is stimulated by inflammatory infiltrates or OS damage, MAPK is rapidly activated. Through intracellular signal cascade amplification, it precisely regulates HSCs biological behavior: on one hand, it drives the transformation of quiescent HSCs into myofibroblasts; on the other hand, it modulates the proliferation-apoptosis balance of HSCs, promoting the massive synthesis and secretion of core ECM components such as type I and III collagen (Ruiz de Galarreta et al., 2023). Concurrently, the activated MAPK upregulates the expression of pro-fibrotic transcription factors, further exacerbating abnormal ECM deposition. This disrupts the homeostatic balance between matrix synthesis and degradation within the liver tissue, ultimately propelling LF from early injury toward irreversible cirrhosis. Inhibiting excessive MAPK activation can block HSCs activation at its source, reduce ECM synthesis and accumulation, thereby achieving delayed or even reversed pathological changes in LF (Parola and Pinzani, 2019). Thus, targeted regulation of MAPK represents a crucial strategy for blocking LF progression, holding significant clinical translational value (Figure 2).

### 3.1 ERK1/2

ERK1/2 is a core member of the MAPK family, transmitting extracellular signals through the RAF-MEK-ERK cascade and serving as a key signaling hub regulating cellular physiological activities (Roskoski, 2012). Under physiological conditions, ERK1/2 participates in core processes such as cell proliferation, differentiation, survival, and metabolic regulation. For instance, it guides directed cell differentiation during embryonic development and maintains cellular homeostasis in adult organisms by regulating cyclin expression. Concurrently, it activates anti-apoptotic proteins to protect cells from stress-induced damage. Its activity is tightly regulated by negative feedback mechanisms, including phosphorylation of upstream molecules or induction of inhibitory factor synthesis, preventing excessive signal activation (Pearson et al., 2001). Under pathological conditions, sustained ERK1/2 activation disrupts cellular equilibrium and contributes to multiple disease states. In tumors, it promotes cancer cell proliferation, migration, and epithelial-mesenchymal transition (Liu et al., 2025). In cardiovascular diseases, excessive activation exacerbates myocardial fibrosis (Luo et al., 2017). This pathologically activated state also plays a pivotal role in LF progression.

The core of LF is the activation of HSCs. Under chronic liver injury, normally quiescent HSCs transform into myofibroblasts that secrete large amounts of ECM, leading to excessive ECM deposition (Bataller and Brenner, 2005). ERK1/2 serves as the pivotal signaling mediator linking liver injury to HSCs activation: Following liver injury, cytokines such as PDGF-BB released by platelets and macrophages bind to receptors on HSCs surfaces, activating the Ras-Raf-MEK pathway and ultimately inducing ERK1/2 phosphorylation and activation. Activated ERK1/2 translocates to the nucleus, phosphorylating transcription factors such as c-Fos and Elk-1. This upregulates expression of fibrosis markers like α-SMA and collagen while suppressing genes involved in ECM degradation, thereby exacerbating matrix deposition (Sharma et al., 2025). Furthermore, ERK1/2 amplifies hepatic inflammatory responses by regulating proinflammatory cytokine secretion, thereby further promoting HSCs activation and establishing a vicious cycle of fibrosis progression. Fang et al. reported that inhibiting ERK1/2 activity significantly reduced HSCs proliferation and ECM synthesis, alleviating the severity of LF (Fang et al., 2025). These studies indicate that ERK1/2 serves as a key signaling molecule mediating HSCs activation and ECM deposition induced by liver injury. Its targetability provides a core direction for investigating the mechanisms of LF and developing precision therapies, laying the foundation for subsequent related research.

### 3.2 p38 MAPK

p38 MAPK, a major subfamily of the MAPK family, transmits extracellular stress and inflammatory signals through the MKK3/6-p38 cascade. It serves as a pivotal signaling hub regulating cellular stress responses, inflammatory reactions, and tissue repair (Denny, 2022). Under physiological conditions, p38 MAPK participates in stress adaptation, inflammatory homeostasis regulation, and tissue damage repair. In response to stimuli such as OS and infection, it activates downstream transcription factors to regulate inflammatory mediator release, maintaining microenvironment equilibrium while mediating cell proliferation and matrix remodeling. Its activity is subject to negative feedback regulation, enabling precise control over signal intensity and duration to prevent cellular dysfunction (Yong et al., 2009). In pathological states, p38 MAPK shifts from controlled transient activation to abnormal sustained activation, disrupting cellular homeostasis and contributing to various disease processes. Notably, within the tumor microenvironment, persistently activated p38 MAPK enhances tumor cell invasion and migration by regulating downstream target gene expression, thereby promoting malignant progression (Wagner and Nebreda, 2009). In pulmonary fibrosis, excessive activation of this pathway accelerates the transformation of pulmonary interstitial cells into myofibroblasts, drives abnormal ECM deposition, and ultimately exacerbates pathological changes associated with pulmonary fibrosis (Ji et al., 2016).

Similarly, in hepatic pathological evolution, p38 MAPK serves as a core driver of LF formation and progression, advancing fibrosis through multidimensional mechanisms (Liu et al., 2023). Following chronic liver injury (e.g., viral infection, alcoholic damage, metabolic abnormalities), hepatocytes and macrophages release inflammatory and growth factors such as TNF-α, IL-1β, and PDGF. These signaling molecules bind to receptors on the surface of HSCs, inducing phosphorylation and activation of p38 MAPK (Ding et al., 2024). Activated p38 MAPK enters the nucleus, where it phosphorylates transcription factors such as ATF2, MEF2, and MK2 transcription factors. This process simultaneously upregulates the expression of fibrosis markers such as α-SMA and type I collagen, driving the transformation of quiescent HSCs into myofibroblasts. Concurrently, it suppresses the expression of genes associated with ECM degradation, such as matrix metalloproteinases (MMPs), leading to an imbalance between ECM synthesis and degradation and exacerbating matrix deposition (Huo et al., 2023). Furthermore, p38 MAPK amplifies local hepatic inflammation by regulating inflammatory cytokine secretion from hepatocytes and Kupffer cells, thereby further promoting HSCs activation and establishing a vicious cycle of “inflammation-activation-fibrosis” (Wang et al., 2022). Previous studies have demonstrated that inhibiting p38 MAPK effectively suppresses HSCs proliferation and ECM synthesis, significantly alleviating the severity of LF. This suggests that this pathway represents a potential therapeutic target for LF (Khalil et al., 2020).

### 3.3 JNK

JNK (c-Jun N-terminal kinase), as a core member of the MAPK family, is a prototypical stress-responsive serine/threonine protein kinase. Its defining characteristic lies in its ability to rapidly detect and respond to diverse external stimuli (Castro-Torres et al., 2024). Whether triggered by regulatory signals from cytokines (such as tumor necrosis factor and interleukins), damage stimuli from ionizing radiation or ultraviolet irradiation, or microenvironmental fluctuations like OS and osmotic pressure changes, JNK can be precisely activated. At the physiological level, JNK initiates the ordered expression of downstream target genes by specifically phosphorylating transcription factors such as c-Jun and ATF2. This regulatory function encompasses all major cellular life processes: it is involved in the morphogenesis and functional maturation of tissues and organs during embryonic development, modulates the orderly progression of the cell cycle to control cell proliferation and differentiation, and exerts a pivotal role in the initiation and execution of apoptosis, thereby sustaining tissue homeostasis and dynamic balance in cellular populations (Semba et al., 2020). However, pathological dysregulation of JNK disrupts its regulatory functions, making it a major driver in the onset and progression of various diseases. Extensive research confirms that abnormal activation or sustained activation of JNK is closely associated with neuronal damage in neurodegenerative diseases such as Alzheimer’s disease and Parkinson’s disease (Zhao et al., 2022). It exerts oncogenic effects in the proliferation, invasion, and metastasis of malignant tumors like lung and liver cancers (Abdelrahman et al., 2021), while also participating in the pathological processes of metabolic disorders such as diabetes and obesity, causing severe disruption to organismal homeostasis (Feng et al., 2020).

Notably, as a core signaling molecule, JNK exhibits tight regulatory interactions with the pathological progression of LF and directly influences its development (Brenner et al., 2013). As the body’s central organ for metabolism and detoxification, the liver is susceptible to damage from various factors including viral infections, drug toxicity, and alcohol exposure. Abnormal activation of JNK represents a critical juncture that disrupts the balance between liver injury and repair, thereby driving the progression of fibrosis (Piccolo et al., 2021). Specifically, during chronic liver injury, sustained JNK activation exacerbates hepatocyte inflammatory apoptosis by modulating inflammatory signaling pathways, leading to massive hepatocyte loss and disruption of hepatic parenchymal architecture. Concurrently, it directly targets quiescent HSCs, inducing their activation and transformation into myofibroblast-like cells through phosphorylation of regulatory proteins. This process also promotes extensive proliferation of activated HSCs (Zhang et al., 2023). Activated HSCs continuously secrete ECM components such as collagen while exhibiting significantly reduced degradation capacity. This ultimately results in excessive deposition and abnormal accumulation of ECM within the liver, progressively replacing normal hepatic tissue. The continuous progression of this process drives the persistent worsening of LF, ultimately leading to cirrhosis and even hepatocellular carcinoma (Yang et al., 2019). Thus, JNK has emerged as a key molecular switch driving the initiation and progression of LF, representing a crucial potential target for targeted intervention in liver diseases.

## 4 Role of MAPK in liver fibrosis

### 4.1 Suppression of inflammatory responses

As a core signaling system regulating hepatic stress responses and inflammatory reactions, abnormal activation of MAPK constitutes the key molecular basis for initiating chronic inflammation in liver tissue, amplifying inflammatory cascades, and driving the progression of fibrosis. It has emerged as a central target area for exploring the pathological mechanisms of LF and developing targeted intervention strategies (Vargas-Pozada et al., 2022). Studies confirm that in liver tissue from animal models of fibrosis, phosphorylation levels of key MAPK family subtypes (p38 MAPK, ERK1/2, JNK) are significantly elevated, accompanied by abnormal overexpression of proinflammatory factors such as IL-6, TNF-α, and TGF-β1 (Liu et al., 2023). These molecular alterations show a significant positive correlation with the staging and severity of LF, suggesting that MAPK may profoundly contribute to the pathological progression of LF by regulating persistent inflammatory responses and HSCs activation.

In interventional studies of LF, strategies targeting MAPK inhibition demonstrate promising therapeutic potential. Their mechanism of action not only involves direct disruption of pro-inflammatory signaling cascades but also effectively remodels the tissue repair microenvironment, thereby mitigating fibrosis progression (Lan et al., 2024). Studies in animal models of LF indicate that specific inhibition of MAPK activity significantly downregulates the transcription and secretion of proinflammatory factors such as TNF-α and IL-6 in liver tissue, reducing inflammatory infiltration and necrotic damage in hepatocytes. Concurrently, it directly suppresses HSCs activation and proliferation, diminishing excessive deposition of ECM components like type I collagen and fibronectin, thereby achieving effective blockade at the core stages of fibrosis progression (Chen J. et al., 2023).

Furthermore, targeted MAPK inhibition exerts indirect anti-inflammatory and anti-fibrotic effects by modulating the hepatic immune microenvironment. Studies reveal that suppressing MAPK regulates the polarization of hepatic macrophages, promoting the conversion of pro-inflammatory macrophages to anti-inflammatory/reparative macrophages. This reduces inflammatory cytokine release while upregulating tissue repair-associated cytokines (Chen et al., 2025). Concurrently, this regulatory effect improves hepatic microcirculatory dysfunction, promotes regeneration and repair of damaged hepatocytes, and facilitates the restoration of the liver’s homeostatic microenvironment.

These studies demonstrate that MAPK-targeting therapeutic strategies, which efficiently block pro-inflammatory and pro-fibrotic signaling while preserving physiological regulatory functions, represent a core breakthrough for future LF treatment research. This approach not only offers a novel therapeutic perspective for breaking the vicious “inflammation-fibrosis” cycle in LF progression but also holds promise for advancing treatment from symptomatic relief toward precise reversal. It provides crucial theoretical support for targeted clinical interventions, enhanced therapeutic efficacy, and improved prognosis in LF.

### 4.2 Inhibition of pathological angiogenesis

Angiogenesis is a highly regulated physiological process during organismal development and tissue repair, playing a crucial role in maintaining normal hepatic blood supply, ensuring nutrient delivery to hepatocytes, and supporting metabolic function. The process of hepatic angiogenesis, particularly in chronic liver diseases such as LF, assumes a pivotal role (Li et al., 2023). Under normal conditions, angiogenesis facilitates tissue repair and functional reconstruction in the liver, maintaining homeostasis within the hepatic microenvironment. However, dysregulated angiogenesis leads to excessive proliferation of pathological vessels within the liver, disrupting microenvironmental equilibrium. This triggers activation of HSCs, excessive ECM deposition, and exacerbates the progression of LF (Zhao et al., 2012). During the pathological process of LF, liver tissue is subjected to multiple stresses including persistent inflammatory infiltrates, OS, and hepatocyte injury, leading to abnormal activation of pathological angiogenesis. As a key intracellular signaling network, MAPK influences the fate of pathological vascular formation in LF by regulating the expression and activation of downstream angiogenesis-related molecules, making it a significant potential therapeutic target (Qiao et al., 2020). Reports indicate that abnormal activation of MAPK in animal models of LF exacerbates hepatic inflammation and excessive pathological angiogenesis. It also increases the expression of the angiogenesis-related protein VEGF, promoting vascular endothelial cell proliferation, migration, and lumen formation, thereby worsening the severity of LF (Deng et al., 2023).

In summary, MAPK plays a crucial role in regulating pathological angiogenesis, particularly as a key pathological mechanism in the onset and progression of LF. Targeting MAPK and its associated angiogenic factors can effectively inhibit pathological vascular proliferation within the liver, mitigate pathological damage caused by LF, and delay disease progression. Inhibitors targeting MAPK offer a novel therapeutic strategy by modulating pathway activity to suppress pathological angiogenesis, thereby providing potential targets for the clinical treatment of LF.

### 4.3 Alleviating oxidative stress

Oxidative stress (OS) fundamentally represents a pathological state of imbalance between the body’s oxidative and antioxidant systems. Specifically, it manifests when the production rate of reactive oxygen species (ROS) exceeds the clearance capacity of the body’s antioxidant system, leading to cellular and tissue oxidative damage upon excessive ROS accumulation (Sadasivam et al., 2022). This imbalance is particularly pronounced in the pathological progression of LF, where key cellular populations such as hepatocytes and HSCs persistently reside in a high OS microenvironment, resulting in substantial ROS production. These excess ROS not only directly cause structural damage and functional disruption in hepatocytes but also induce HSCs activation and exacerbate local inflammatory responses. Ultimately, they drive abnormal accumulation of the ECM, becoming one of the core drivers of LF progression (Hao et al., 2024).

As a central signaling hub for cellular responses to OS, MAPK profoundly participates in the regulation of LF through a cascade-like signal transduction mechanism (Shi et al., 2016). When ROS levels abnormally surge in liver tissue, key subfamilies of the MAPK family (p38 MAPK, JNK, ERK) undergo rapid activation. The activated MAPK directly influences the synthesis efficiency of core antioxidant enzymes such as SOD and GPx by regulating the expression of multiple downstream target genes (Gu et al., 2016). Notably, MAPK does not solely regulate antioxidant enzyme expression; it also forms a synergistic regulatory network with the Nrf2 signaling pathway. Through signal interaction, this network further enhances the antioxidant reserve capacity of liver tissue and mitigates oxidative damage (Wei et al., 2020).

The above research evidence indicates that targeted intervention strategies centered on MAPK can effectively alleviate local inflammatory responses in the liver, inhibit HSCs activation, reduce abnormal ECM deposition, and promote repair of damaged liver tissue by regulating OS balance. This regulatory approach provides novel theoretical foundations and technical directions for overcoming traditional bottlenecks in LF treatment, holding significant clinical translational value.

### 4.4 Inhibiting hepatocyte activation

HSCs activation serves as a core driver in the progression of LF and is pivotal in the pathological outcomes of liver injury repair. This activation process plays a critical role, particularly in fibrosis induced by chronic liver injuries such as viral hepatitis, alcoholic liver disease, and non-alcoholic fatty liver disease (Kisseleva and Brenner, 2021). Under normal conditions, HSCs remain quiescent, participating in physiological functions such as vitamin A storage while maintaining hepatic microenvironmental homeostasis and metabolic equilibrium. However, abnormal activation leads to their proliferation and transformation into myofibroblasts, resulting in excessive secretion of ECM components like collagen. This forms fibrous scar tissue that disrupts normal liver structure and function, propelling fibrosis progression toward cirrhosis and even hepatocellular carcinoma (Deng et al., 2024). During the pathological process of LF, the liver tissue is continuously subjected to multiple stimuli including inflammatory infiltration, OS, and cytokine dysregulation, leading to the abnormal initiation of the HSCs activation program (Parola and Pinzani, 2019). MAPK dominates the activation fate of HSCs by regulating the expression and activation of downstream molecules involved in proliferation, differentiation, and fibrosis, making it a key potential therapeutic target for LF. Notably, in CCl_4_-induced mouse models of LF, abnormal activation of MAPK significantly promotes hepatocyte activation and proliferation. This pathway upregulates the expression of fibrosis marker proteins such as α-SMA and Colla1, accelerates ECM deposition, and thereby exacerbates the severity of LF (Liu N. et al., 2019).

These studies demonstrate that MAPK plays a crucial role in regulating HSCs activation, establishing a key pathological mechanism in the onset and progression of LF. Targeting MAPK and its associated regulators of HSCs activation can effectively inhibit the fibrotic process and improve liver function. Specific MAPK inhibitors or natural bioactive compounds block MAPK activation to suppress HSCs activation, offering a novel intervention strategy for LF and providing a highly promising targeted approach for its clinical treatment.

### 4.5 Inhibiting excessive extracellular matrix deposition

The dynamic equilibrium of the ECM is fundamental to maintaining normal liver structure and function, playing a crucial role in safeguarding the hepatocyte survival microenvironment and regulating hepatic metabolism and repair processes. During LF progression, metabolic imbalance of the ECM, particularly excessive deposition, serves as a core driving factor (Zuo et al., 2023). Under normal physiological conditions, ECM synthesis and degradation maintain a precisely regulated dynamic equilibrium, simultaneously providing structural support for hepatocytes and participating in post-injury repair and reconstruction. However, abnormal ECM deposition leads to fibrotic remodeling of liver tissue, disrupting hepatic microcirculation and progressively causing liver function decline. If unchecked, this progression may further develop into cirrhosis or even hepatocellular carcinoma (Villesen et al., 2020). During the pathological process of LF, the liver tissue remains in a state of chronic inflammatory infiltration, disrupted hepatocyte repair mechanisms, and massive release of pro-fibrotic factors. This leads to abnormal activation and transformation of HSCs into myofibroblasts, which become the primary source of ECM synthesis. MAPK as a core intracellular signaling hub, regulates the expression and activation of downstream pro-fibrotic molecules, thereby influencing the activation state of HSCs and the balance between ECM synthesis and degradation. Consequently, it emerges as a significant potential therapeutic target for LF (Yang J. et al., 2023). Reports indicate that in carbon tetrachloride-induced LF mouse models, abnormal activation of MAPK significantly promotes the activation and proliferation of HSCs. This pathway upregulates the expression of ECM components such as collagen I, collagen III, and fibronectin, while simultaneously suppressing the activity of matrix metalloproteinases. Consequently, it reduces the efficiency of ECM degradation and accelerates the progression of LF (Shi et al., 2016).

In summary, MAPK plays a crucial role in regulating ECM metabolic balance. Targeting MAPK and its associated pro-fibrotic factors can effectively inhibit HSCs activation, reduce excessive ECM deposition, thereby alleviating the severity of LF and delaying disease progression. Therefore, targeting MAPK to inhibit excessive ECM deposition offers a novel therapeutic strategy for LF, providing a potential effective target for its clinical treatment.

## 5 Natural drugs targeting the MAPK signaling pathway for liver fibrosis treatment

**FIGURE 3:**
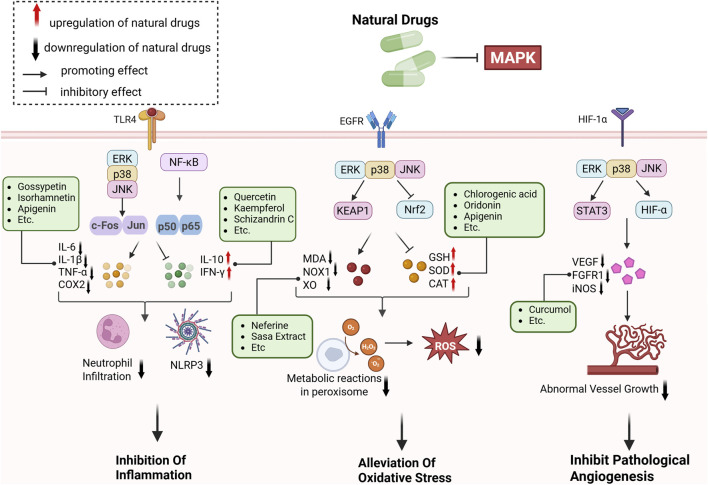
Mechanisms by which natural drugs target MAPK to treat LF through inhibiting inflammation, alleviating oxidative stress, and suppressing pathological angiogenesis.

**FIGURE 4:**
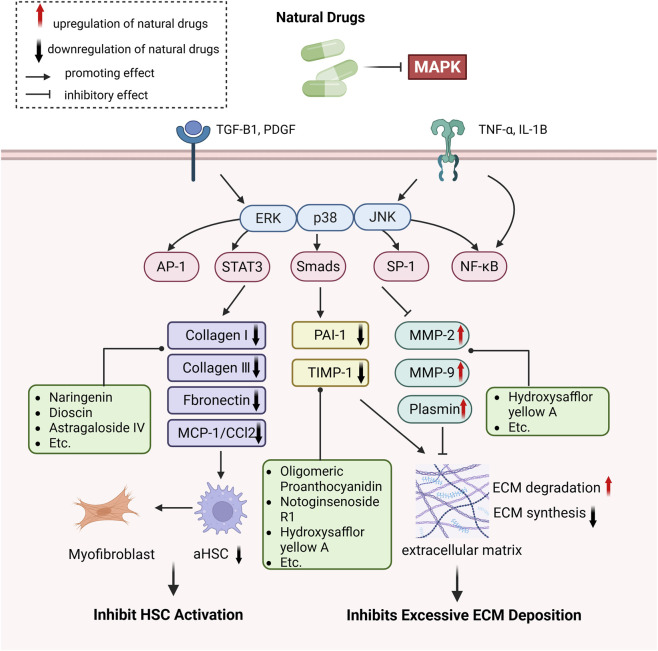
Mechanisms by which natural drugs target MAPK to treat LF through inhibiting HSCs activation and suppressing excessive ECM deposition.

Numerous studies confirm that natural drugs such as flavonoids, terpenoids, and alkaloids can target MAPK. By regulating the activation state of this pathway, they maintain the balance of the liver tissue microenvironment and improve hepatocyte physiological functions. This regulatory effect alleviates hepatic inflammatory infiltration, repairs pathological damage, suppresses the secretion of pro-inflammatory and fibrosis-related cytokines, inhibits abnormal activation and proliferation of HSCs, and reduces excessive ECM deposition. Concurrently, the liver’s antioxidant stress potential is effectively stimulated. Consequently, these natural drugs demonstrate significant application potential in the therapeutic field of LF (Table 1; Figures 3, 4).

**TABLE 1:** Natural drugs regulate MAPK signaling pathway to treat hepatic fibrosis.

Extract	*Origination*	Structure	Dosage	Model	Biological effects	Results	Limitations	References
Quercetin	*Taxus wallichiana Zucc.*	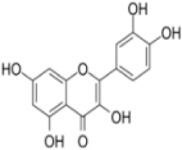	5, 15 mg/kg	CCl_4_-induced mice	TBIL, ALT, AST↓ HA, LN, IV-C, PIIIP↓ NF-κB, p38 MAPK↓ Bcl-2↑Bax, caspase-3↓ TNF-α, IL-6, IL-1β, Cox-2, TGF-β↓	Improve pathological morphological changes anti-inflammatory Anti-apoptotic	No positive control group was established	Wang et al. (2017)
Oligomeric proanthocyanidin	*Vitis vinifera L.*	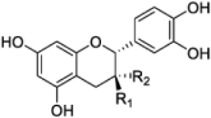	5, 10, 20, 40 μg/mL	HSC-T6 cells	CollagenI, α-SMA, TIMP-1↓ JNK/ERK MAPK, PI3K/Akt, NF-κB↓	Inhibit the transdifferentiation Inhibit HSCs activation	No animal model of liver fibrosis has been established	Jiang et al. (2017)
Isorhamnetin	*Hippophae rhamnoides L.*	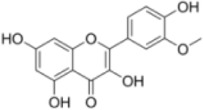	10, 30 mg/kg	CCl_4_-induced mice BDL-induced mice	ALT, AST↓ PPAR-γ↑ Beclin-1, LC3, TGF-β1↓ Smad3/p38 MAPK↓	Improve the pathophysiological manifestations Inhibit autophagy	Other mechanisms of action have not been investigated, and the safety of clinical application remains to be verified	Liu Y. et al. (2019)
Naringenin	*Citrus medica L.*	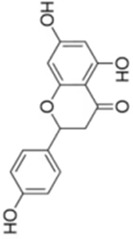	100 mg/kg	HFD-induced mice CCl_4_-induced mice	ALT, AST↓ TG, TC↓ α-SMA, Col-1, Col-3↓ Bax, Caspase3, Caspase7↑ Bcl2↓ p-JNK, p-ERK, FoxO3a↓	Inhibit the inflammatory response and collagen fiber deposition Promote apoptosis of activated HSCs Maintain hepatocyte survival	No concentration gradient has been set.	Ma et al. (2024)
			100, 200 μM	LX2 cells				
Kaempferol	*Ginkgo biloba L.*	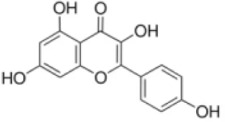	50, 100 mg/kg	CCl_4_-induced mice	ALT, α-SMA, Col1a1↓ iNOS, TNF-α, IL-6↓ MAPK/NF-κB↓	Regulate macrophage polarization	The effects of KA on M2 macrophage polarization have not been thoroughly investigated	Chen et al. (2025)
Gossypetin	*Rhododendron dauricum L.*	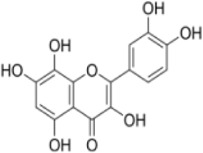	10 mg/kg	CCl_4_-induced mice	ALT, AST↓ MKK3/6-p38 MAPK↓ p53, IL-1α, TNF-α↓ IL-10↑	Ameliorate anxiety- and depression-like behaviors and cognitive impairments Maintain the homeostasis of the central nervous system	Safety of the drug has not been evaluated	Xu et al. (2024)
			40 μM	LX2 cells				
Didymin	*Clinopodium polycephalum (Vaniot) C.Y.Wu and S.J.Hsuan*	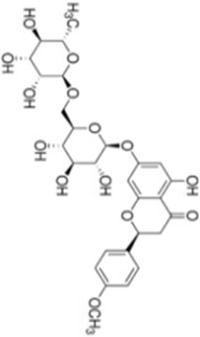	0.5 mg/kg	CCl_4_-induced mice	ALT, AST, TNF-α↓ ERK/MAPK, PI3K/Akt↓ Bcl-2, cyclin B1, cyclin D1↓ CDK4↓ Hyp, PCIII↓ Bax, caspase-3/9↑ RKIP↑	Alleviate chronic liver injury and collagen deposition Improve the pathological disorder of hepatic tissue Induce apoptosis Arrest cell cycle at the G2/M phase	Long-term toxicity and drug combination synergistic effects have not been evaluated	Lin et al. (2016)
			50, 25, 12.5, 6.25, 3.125 μM	HSC-T6 cells				
Grape seed proanthocyanidin	*Vitis vinifera L.*	——	10, 25 μg/mL	LX-2 cells	IL-1β, IL-6, IL-8↓ iNOS, COX-2, IκBα↓ NF-κB p65, MAPK, Akt↓	Anti-inflammatory	Based solely on *in vitro* cell models, without involving *in vivo* animal experiments or human clinical trials	Lee et al. (2017)
Apigenin	*Matricaria recutita L.*	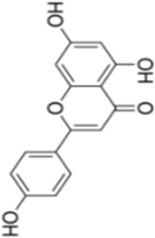	100, 300, 600 mg/kg	Wistar mice	AST, ALT↓ MDA, ECM↓ TGF-β1, α-SMA↓ ALB, TP↑ SOD, GSH-PX↑	Alleviate hepatic necrosis Alleviate inflammatory infiltration Alleviate collagen deposition Alleviate oxidative stress	Lack of pharmacokinetic data support	Qiao et al. (2020)
Dioscin	*Dioscorea nipponica Makino*	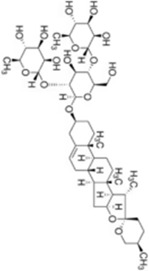	20, 40, 60 mg/kg	BDL-induced mice DMN-induced mice	ALT, AST↓ TBIL↓ α-SMA, COL1A1, COL3A1↓ MDA↓ ROS↓ p38 MAPK↓ GSH, SOD↑ HO-1, GST↑	Inhibit oxidative stress, block HSCs activation Inhibit collagen deposition	The experimental design for mechanism validation is one-sided and lacks the depth of reverse validation	Gu et al. (2016)
			0.6, 1.2, 2.4 μg/mL	HSC-T6/LX2 cells				
Astragaloside IV	*Astragalus mongholicus Bunge*	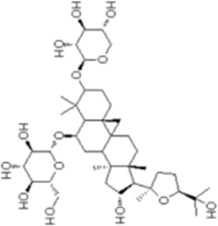	3, 10, 30, 100 μM	HSCs	ROS↓ LPO↓ α-SMA, Col I↓ Fibronectin↓ p38 MAPK↓ GSH↑ Nrf2↑ GSH↑	Alleviate oxidative stress Reduce collagen secretion	Has not yet been systematically validated in animal models or clinical samples	Li et al. (2013)
Notoginsenoside R1	*Panax notoginseng (Burk) F. H. Chen*	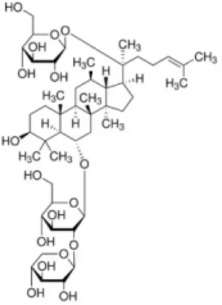	25, 50, 100 mg/kg	CCl_4_-induced mice	ALP, AST, ALT↓ Coll-a1, α-SMA ↓ TIMP1↓ MDA↓ IL-1β, IL-6, TNF-α↓ p-p65, p-ERK, p-JNK, p-p38↓ ALB↑ TP↑ PPAR-γ↑ GSH, SOD, GST↑	Ameliorate pathological damage Inhibit HSCs activation Alleviate oxidative stress Inhibit the inflammatory response	The direct molecular target through which this compound exerts its effects has not been identified	Gong et al. (2022)
Hydroxysafflor yellow A	*Carthamus tinctorius L.*	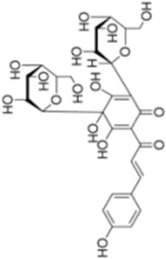	10 mg/mL	CCl_4_-induced mice HFD-induced mice	HA, LN↓ TIMP-1, TGF-β1↓ p38 MAPK↓ PPAR-γ, MMP-2↑	Inhibit the inflammatory response Inhibit hepatic fibrosis Improve liver function indicators	No drug concentration gradient has been established	Liu et al. (2014)
NPLC0393	*Gynostemma pentaphyllum (Thunb.) Makino*	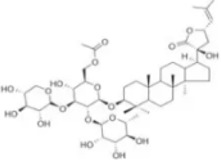	5 mg/kg	CCl_4_-induced mice	ERK↓ JNK↓ α-SMA↓ COL1A1↓ ECM↓ TGF-β1↓ NDRG2↑	Inhibit HSCs activation Inhibit hepatic fibrosis	No drug concentration gradient has been established	Huang et al. (2020)
4-Methoxysulfonylpaeonol	*Paeonia suffruticosa Andrews*	——	5 mg/kg	CCl_4_-induced mice	TGF-β1/Smad↓ PDGF-BB/MAPK↓ Akt↓ α-SMA, col1A2↓	Ameliorate hepatic tissue injury Reduce collagen deposition	Long-term toxicity, safety margin, and metabolic mechanisms *in vivo* have not been investigated	Liao et al. (2020)
			0–20 μM	LX2cells/HSC-T6cells				
Dehydroginger flavone	*Zingiber officinale Roscoe*	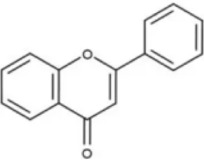	25, 50 mg/kg	TAA-induced mice	AST, ALT↓ ALP↓ α-SMA, COL1A1, COL3A1↓ MPO, NE↓ p38, ERK↓ α-SMA, FN1, TIMP3↓	Inhibit the inflammatory response Inhibit HSCs activation Alleviate oxidative stress	The clinical translational efficacy of DHZ has not been investigated	Sharma et al. (2022)
			12.5–50 μM	HSC-LX2 cells				
Chlorogenic acid	*Lonicera japonica Thunb.*	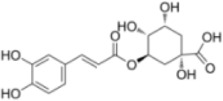	60 mg/kg	CCl_4_-induced mice	α-SMA↓ TIMP-1↓ MDA↓ CYP2E1↓ p47phox↓ gp91phox↓ ROS↓ p38↓ ERK1/2↓ GSH↑ SOD, CAT↑	Inhibit hepatic fibrosis Reduce collagen deposition Inhibit HSC proliferation	The drug concentration gradient has not been investigated	Shi et al. (2016)
			12.5, 25, 50 μg/mL	HSC-T6 cells				
Curcumin	*Curcuma longa L.*	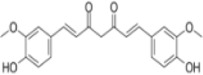	400 mg/kg	TAA-induced mice	MAT2B↓ p38 MAPK↓ Collagen α1↓	Inhibit hepatic fibrosis Inhibit HSC proliferation Alleviate oxidative stress	No positive drug group was added	Hu and Zhou (2020)
			20 μM	HSC cells				
Carvacrol	*Origanum vulgare L.*	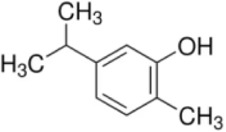	25, 50, 100 mg/kg	CCl_4_-induced mice	ALT, AST↓ α-SMA, Col1α1↓ ERK1/2↓ JNK1/2↓ p38↓	Ameliorate hepatic tissue injury Reduce collagen production	Long-term toxicity and pharmacokinetic studies of carvacrol have not been conducted	Cai et al. (2021)
			100, 300, 500 μM	HSC-T6 cells				
Betulinic acid	*Ziziphus jujuba Mill.*	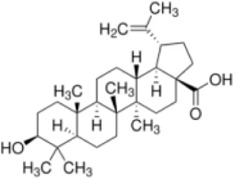	15, 30, 60 mg/kg	CCl_4_-induced mice	ALT, AST↓ Hyp, PDGF-BB↓ α-SMA↓ COL-1↓ LC3B-II, ATG7↑ MAPK/ERK↓	Ameliorate hepatic tissue injury Induce autophagy	Knockout models have not been investigated, and *in vitro* mechanism validation has not been thoroughly conducted	Liu N. et al. (2019)
			40 μM	HSC cells				
Oridonin	*Rabdosia rubescens (Hemsl.) Hara*	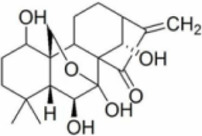	5–40 μM	HSC-T6	subG1, Caspase 3↑ GSH↓ ROS↑ ERK, JNK, p38 MAPK↑	Induce cell apoptosis Alleviate oxidative stress	The differential effects of different concentrations of oridonin on normal hepatocytes and hepatic stellate cells have not been investigated	Kuo et al. (2014)
Tanshinone IIA	*Salvia miltiorrhiza Bunge*	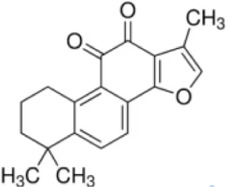	0–30 μM	HSC-T6 cells	α-SMA↓ AKT↓ Bcl-2↓ prohibitin↑ MAPK↑ ERK↑ Bax↑ caspase-3, Caspase-9↑ PARP cleavage↑	Inhibit cell proliferation Ameliorate hepatic tissue injury Induce cell apoptosis	Lack of organoid and humanized model validation and preclinical pharmacokinetic assessment	Pan and Wang (2012)
			10 mg/kg	DMN-induced rats				
Curcumol	*Curcuma phaeocaulis Valeton*	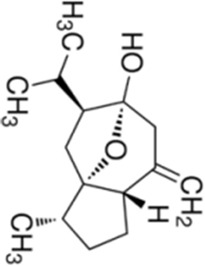	30 mg/kg	CCl_4_-induced mice	Angiopoietin 2↓ VEGFR-2↓ ALT, AST↓ HA, LN↓ KLF5↓ ROS↓ ROS/ERK↓ MDA↓ GSH/GSSG↑	Alleviate tissue inflammatory infiltration Alleviate collagen deposition Enhance cellular antioxidant stress capacity Inhibit hepatic angiogenesis	No preclinical pharmacokinetic or toxicological studies have been conducted	Gao et al. (2021)
Mogroside IVE	*Siraitia grosvenorii (Swingle) C.*	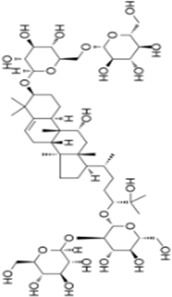	25 mg/kg	CCl_4_ induced mice	α-SMA, Col I↓ HIF-1α↓ ALT, AST↓ TLR4/MyD88↓ MAPK↓	Inhibit HSC proliferation Inhibit the inflammatory response Alleviate collagen deposition	Acute liver fibrosis model induced solely by CCl_4_	Cao et al. (2018)
			10 μM	HSC-T6 cells RAW264.7 cells				
Schizandrin C	*Schisandra chinensis (Turcz.) Baill.*	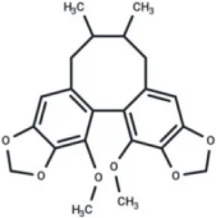	50 mg/kg	CCl_4_-induced mice	ALT, AST↓ TBIL↓ α-SMA↓ IL-6, TNF-α↓ p38/ERK MAPK↓ NF-κB↓	Improve hepatic tissue structure Inhibit HSCs activation Inhibit hepatic fibrosis	A single-dose intervention was used, and the dose-response relationship was not clearly established	Chen P. et al. (2023)
			20, 40, 80 μM	LX-2/HSC-T6 cells				
Schisantherin A	*Schisandra chinensis (Turcz.) Baill.*	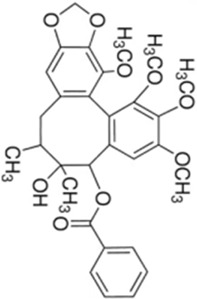	1, 2, 4 mg/kg	CCl_4_-induced mice	α-SMA, COL1A1↓ Hyp↓ TAK1/MAPK↓ NF-κB↓ TNF-α, IL-1β, IL-6↓	Inhibit hepatic fibrosis Ameliorate hepatic tissue pathological disorder Alleviate collagen deposition Inhibit the inflammatory response	No pharmacokinetic studies, liver-targeted distribution studies, or long-term toxicity assessments have been conducted	Wang H. et al. (2021)
			0.625–5 μM	HSC-T6/RAW264.7 cells				
Neferine	*Nelumbo nucifera Gaertn.*	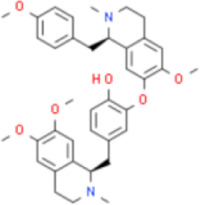	5, 10 mg/kg	CCl_4_-induced mice	ALT, AST, TBIL↓ HA, PIIINP, IV-C, LN↓ MDA↓ p38 MAPK, ERK1/2, JNK↓ α-SMA, TGF-β1↓ TNF-α, IL-6↓ SOD↑ GSH-PX, CAT↑	Inhibit hepatic fibrosis Alleviate oxidative stress Inhibit the inflammatory response	The dose-response characteristics were not thoroughly analyzed, and the dose dependency of some indicators remains unclear	Wang Y. et al. (2021)
Ophiopogonis Radix	*Ophiopogon japonicus (L. f) Ker-Gawl.*	——	50, 100 mg/kg	CCl_4_-induced mice	ALT, AST↓ Hyp↓ HA, COL IV↓ TGF-β/Smads↓ α-SMA, COL1A1↓ p38, ERK, JNK↓ IL-1β, IL-6↓ ATF4/Caspase 3↓	Ameliorate hepatic tissue injury Alleviate collagen deposition Inhibit cell apoptosis	The structure-activity relationship of OJP-W2 has not been thoroughly investigated, and the molecular mechanisms underlying the differences in effects among different dosage groups remain unclear	Liu et al. (2024)
Salvianolic acid B	*Salvia miltiorrhiza Bunge*	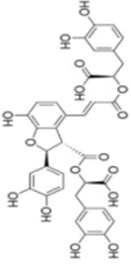	15, 30 mg/kg	DEN-induced mice	α-SMA, Collagen I, TGF-β↓ ERK, JNK, p38↓ P- Smad2L/3L↓ PAI-1↓P-Smad3C↑	Inhibit hepatic fibrosis Inhibit HSCs activation	Lack of validation across different etiologically induced liver fibrosis models	Liu et al. (2024)
			25, 50, 100 μM	LX-2/HSC-T6 cells				
Total polyphenol glycoside extract	*Phlomoides rotata (Benth. ex Hook.f.) Mathiesen*	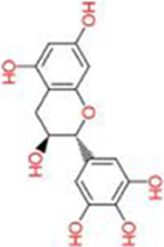	25–50 μg/mL	LX-2 cells	α-SMA↓ Desmin↓ AP1, NF-κB↓ ALT, AST↓ TNF-α, IL-6↓ RAS/MAPK/NF-κB↓ ETS1, GATA4↑	Inhibit hepatic fibrosis Ameliorate hepatic tissue injury Alleviate collagen deposition	The active contributions of the 14 major components and monomer-specific targeting validation remain unidentified	Yang et al. (2024)
			0.05–0.2 g/kg	CCl_4_-induced mice				
Lamiophlomis herba	*Phlomoides rotata (Benth. ex Hook.f.) Mathiesen*	——	800 μg/mL	HSC-T6	α-SMA, PC-III, Col-IV, LN↓ Fibronectin, Col1a1, Col3a1↓ PTGS2, EGFR, SRC↓ MAPK, EGFR/SRC↓	Alleviate oxidative stress Inhibit the inflammatory response Inhibit HSCs activation	*In vitro* cell models fail to fully replicate the complex pathological microenvironment *in vivo*	Chen P. et al. (2023)
Ginkgo biloba extract	*Ginkgo biloba L.*	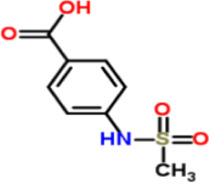	15, 30 mg/kg	DEN-induced mice	p38 MAPK, IκBα↓ NF-κBp65↓ Bax, Caspase-3↓ Bcl-2↑	Inhibit hepatic fibrosis Ameliorate hepatic tissue injury Inhibit the inflammatory response	The specific active ingredient playing a core role in GBE has not been identified	Wang et al. (2015)
Cichorium pumilum Jacq ethyl acetate extract	*Cichorium pumilum Jacq*	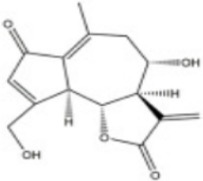	100, 150 mg/kg	TNBS-induced mice	AST, γ-GT↓ IL-6↓ p38, ERK, AKT↓ iNOS, COX-2↓ IL-6, NO↓	Alleviate hepatic congestion Alleviate tissue injury Alleviate colonic ulcers Regulate intestinal flora	The key molecular targets for lactucin’s action remain unidentified	Han et al. (2021)
			12.5, 25, 50 μmol/L	RAW264.7 cells				
Sasa extract	*Sasa veitchii (Carrière) Rehder*	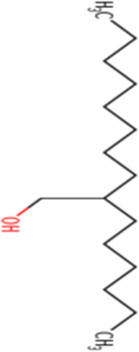	0.1 mL	CCl_4_-induced mice	ALT, AST↓ TNF-α↓ MDA↓ α-SMA↓ NF-κB p65↓ p38, ERK1/2, JNK↓ GSH↑	Inhibit HSCs activation Alleviate hepatic tissue necrosis Alleviate collagen deposition Block oxidative stress Block inflammatory response	Only a single mouse strain and chemically induced model were used, and no *in vitro* experiments were conducted for validation	Yoshioka et al. (2018)
Pomegranate peel extract	*Punica granatum L.*	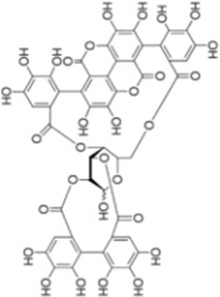	150 mg/kg	CCl_4_-induced mice	ALT, AST↓ T-Bil↓ MDA↓ TGF-β, collagen1-α2↓ α-SMA↓ p38MAPK↓ GSH-Px ↑ Nrf2↑ HO-1, Nqo1↑	Improve liver function Reduce the area of hepatic fibrosis Alleviate oxidative stress Alleviate collagen deposition	Lack of long-term safety data for the drug	Wei et al. (2020)

### 5.1 Flavonoid compounds

As widely distributed bioactive components among plant-derived secondary metabolites, flavonoids exert their remarkable pharmacological activities mainly due to the structural characteristics of the benzopyranone parent nucleus in their molecules and the modification effects of different substituent groups. Notably, these compounds exhibit multiple properties including anti-inflammatory, antioxidant, and anti-HSCs activation effects, demonstrating promising therapeutic prospects in the treatment of LF.

Quercetin is a natural flavonoid compound widely present in various plants, such as *Taxus wallichiana Zucc* (*Taxaceae*). Due to its excellent anti-inflammatory and antioxidant properties, it is commonly used as a nutritional supplement (Alizadeh and Ebrahimzadeh, 2022). Research by Wang et al. demonstrated that quercetin exhibits significant anti-fibrotic effects in a CCl_4_-induced rat LF model. It effectively improved pathological morphological alterations in the liver, significantly reduced levels of liver injury markers such as TBIL, ALT, and AST, and simultaneously decreased serum expression of fibrosis-related indicators including hyaluronic acid (HA), laminin (LN), type IV collagen (IV-C), and procollagen III peptide (PIIIP). Furthermore, quercetin dose-dependently inhibited IκBα degradation, thereby blocking NF-κB pathway activation. Quercetin reduced p38 MAPK phosphorylation to abrogate its signaling, modulated the Bcl-2/Bax apoptotic axis by elevating Bcl-2 expression and reducing Bax expression and caspase-3 activity, and concomitantly regulated the expression of inflammatory mediators (TNF-α, IL-6, IL-1β, Cox-2, TGF-β) and HSCs activation markers (α-SMA, Col1a1, Col1a2, TIMP-1, MMP-1). This suggests that quercetin may exert dual effects by synergistically regulating the NF-κB/IκBα and p38 MAPK anti-inflammatory pathways alongside the Bcl-2/Bax anti-apoptotic pathway, thereby inhibiting hepatic inflammatory responses and hepatocyte apoptosis to delay LF progression (Wang et al., 2017). However, while this study systematically elucidated quercetin’s multi-pathway synergistic regulation of LF-related signaling, it has not yet thoroughly verified whether synergistic molecular networks or reverse feedback regulatory loops exist among these pathways. More refined molecular regulatory mechanisms remain to be further explored.

Oligomeric proanthocyanidins (OPC) are flavonoid compounds widely present in *Vitis vinifera L.* *(Vitaceae)*, exhibiting potent antioxidant activity and diverse pharmacological effects, including free radical scavenging, cardiovascular protection, and improvement of obesity-related metabolic disorders (Gu et al., 2025). OPC pretreatment significantly inhibits LF by reducing tissue fibrosis severity. Jiang et al. demonstrated that OPC (5, 10, 20, 40 μg/mL) concentration-dependently suppressed expression of fibrosis markers (Collagen I, α-SMA, TIMP-1) in LPS-induced HSC-T6 cells while maintaining cellular proliferation balance. Furthermore, OPC inhibited the phosphorylation activation of LPS-stimulated JNK/ERK MAPK and PI3K/Akt pathways, thereby blocking NF-κB translocation from the cytoplasm to the nucleus and ultimately suppressing HSCs transdifferentiation and activation (Jiang et al., 2017). However, despite OPC’s significant anti-fibrotic potential, its *in vivo* pharmacokinetic properties and targeted delivery efficiency remain incompletely characterized, with limited research available. Novel delivery strategies such as nanocarrier technology and drug modification hold promise for enhancing OPC’s bioavailability and hepatic targeting, providing crucial support for its clinical translation.

Isorhamnetin (IH), a flavonol glycoside isolated from *Hippophae rhamnoides L.*, *(Elaeagnaceae)* exhibits diverse pharmacological activities including anti-inflammatory, antioxidant, and antitumor effects (Gong et al., 2020). Liu et al. investigated the potential therapeutic effects and molecular mechanisms of IH in alleviating LF using two mouse models: CCl_4_-induced and bile duct ligation (BDL)-induced LF. Experimental results demonstrated that IH (10, 30 mg/kg) significantly improved pathophysiological manifestations associated with LF. This included reduced ALT and AST levels, decreased hydroxyproline content in liver tissue, and ameliorated pathological alterations such as inflammatory infiltration, hepatocyte swelling and necrosis, and collagen fiber deposition. IH treatment significantly reduced α-SMA expression, upregulated PPAR-γ expression, and reduced excessive deposition of fine ECM components such as Col-1 by regulating the balance between MMP-2 and TIMP1. Additionally, IH significantly decreased Beclin-1 and LC3 expression, suppressed hepatic autophagy levels, and inhibited activation of downstream Smad3 and p38 MAPK by downregulating TGF-β1 expression (Liu N. et al., 2019). *In vitro* experiments further validated IH’s regulatory effects on HSCs activation, ECM formation, and autophagy processes. This work yields critical experimental evidence for the translational potential of IH in LF prophylaxis and therapy, and uncovers its anti-fibrotic mode of action through the suppression of TGF-β1-mediated Smad3/p38 MAPK-dependent autophagy. These findings suggest IH holds promise as a novel therapeutic candidate for LF.

Naringenin, a flavonoid abundant in citrus fruits *Citrus medica L.* *(Rutaceae)*, exhibits anti-inflammatory and antioxidant bioactivities with broad application prospects in liver disease prevention and treatment (Motallebi et al., 2022). Ma et al. investigated its intervention effects and molecular mechanisms in MASH fibrosis through *in vivo* and *in vitro* experiments. *In vivo* experiments employed an HFD + CCl_4_-induced MASH fibrosis model and a BDL-induced LF model. Results showed that naringenin (100 mg/kg/day) significantly improved abnormal liver weight in model mice, reduced serum ALT and AST levels, decreased TG and TC deposition in liver tissue, suppressed inflammatory responses and collagen fiber deposition, and downregulated mRNA and protein expression of α-SMA, Col-1, and Col-3. *In vitro* experiments demonstrated that naringenin (100, 200 μM) inhibited OA + LPS-induced activation of the HSC cell line LX2 by promoting apoptosis through upregulating Bax and Caspase3/7 while downregulating Bcl2. Simultaneously, it suppressed apoptosis in the hepatocyte line LO2 to maintain cell survival. Mechanistic studies indicated that naringenin bound to the active pocket of TAK1 and inhibited its phosphorylation, thereby exerting anti-fibrotic effects through the regulation of the TAK1/MAPK/FoxO3a signaling axis (Ma et al., 2024). However, this study has limitations. It did not thoroughly investigate the pharmacokinetic characteristics, bioavailability, and metabolic pathways of naringenin within the MASH pathological microenvironment. Furthermore, the intervention effects and dose-response relationships across different stages of LF remain unclear. These aspects require further research to elucidate and advance clinical translation.

Kaempferol (KA) is a flavonoid polyphenolic compound widely present in various edible plants such as *Ginkgo biloba L*. *(Ginkgoaceae)* Previous studies have confirmed its diverse pharmacological activities, including anti-inflammatory, antioxidant, and antitumor effects (Dabeek and Marra, 2019). Chen et al. found that KA (50–100 mg/kg) significantly alleviated liver pathological damage in CCl_4_-induced fibrotic mice, reduced ALT levels, decreased macrophage recruitment and collagen deposition in liver tissue, and downregulated expression of fibrosis-related genes and proteins such as α-SMA and Col1a1. Mechanistically, KA suppressed the expression of inducible iNOS, a marker of M1 polarization, in hepatic tissue and LPS + IFN-γ-stimulated THP-1-derived macrophages, concomitantly reducing the secretion of proinflammatory cytokines (TNF-α, IL-6). Combined network pharmacology, molecular docking and Western blot assays further demonstrated that KA bound to ASK1, and it mediated the regulation of macrophage polarization via the inhibition of MAPK/NF-κB activation (Chen et al., 2025). However, this study has certain limitations: it did not thoroughly explore the effects of KA on M2 macrophage polarization or the balanced mechanisms of macrophage phenotype conversion. Future studies should expand sample sizes, employ multi-model validation, and delve into the molecular networks linking KA with gut microbiota and HSCs to provide more robust experimental evidence for clinical translation.

Gossypetin (GTIN), a natural hexahydroxyflavone compound from *Rhododendron dauricum L.* *(Ericaceae)*, exhibits antioxidant, anti-inflammatory, hepatoprotective, and neuroprotective effects. It targets the liver-brain axis to improve LF and central nervous system inflammation, offering protection against chronic liver disease and its neurological complications (Naidoo and Khathi, 2023). In a CCl_4_-induced mouse model of LF, short-term intraperitoneal injection of GTIN (10 mg/kg/day for 7 days) improved delayed weight gain and liver function impairment (reduced ALT and AST activity) while alleviating anxiety-depression-like behaviors and cognitive deficits, suggesting its potential therapeutic value. Mechanistically, GTIN inhibits HSCs and KC activation by suppressing MKK3/6-p38 MAPK activation and downregulating p53 expression, thereby reducing matrix deposition such as Col1a1. It modulates the hepatic inflammatory microenvironment (decreasing IL-1α and TNF-α, increasing IL-10) and enhances antioxidant capacity to reverse HSCs activation. It also ameliorates neuroinflammation in the hippocampus by suppressing activation of microglia (Iba1-positive) and GFAP-positive cells without affecting neuronal numbers. GTIN offers a novel therapeutic strategy for related conditions (Xu et al., 2024). However, this study did not systematically evaluate the long-term safety, pharmacokinetic characteristics, or clinical translational value of GTIN administration. Further in-depth research is required to determine its safety for long-term intervention in patients with chronic LF.

Didymin is a natural flavonoid compound isolated from the citrus fruit *Clinopodium polycephalum (Vaniot) (Lamiaceae) C.Y.Wu and S.J.Hsuan*, exhibiting potential anti-inflammatory and hepatoprotective pharmacological activities (Lv et al., 2024). In a CCl_4_-induced rat LF model, Didymin (0.5 mg/kg) significantly reduced ALT and AST activity as well as TNF-α levels, alleviated chronic liver injury and collagen deposition, and improved hepatic tissue pathological disorders. Its mechanism involves regulating RKIP expression and inhibiting the activation of ERK/MAPK and PI3K/Akt. *In vitro* experiments demonstrated that within a concentration range showing no significant cytotoxicity (≤100 μmol/L), Didymin dose- and time-dependently inhibited the proliferation of HSC-T6 cells, induced apoptosis, and arrested the cell cycle at the G2/M phase. It reduced the expression of Bcl-2, cyclin B1, cyclin D1 and CDK4, and elevated Bax expression. It also disrupted mitochondrial membrane potential, facilitated cytochrome c release and increased caspase-3/9 activity, while abrogating LPS-induced inflammatory responses in RAW264.7 macrophages and decreasing the expression of collagen-associated markers such as hyaluronic acid and PCIII (Lin et al., 2016). These effects are attributed to Didymin’s upregulation of RKIP expression, which modulates ERK/MAPK protein phosphorylation and PI3K/Akt expression, demonstrating multi-targeted anti-fibrotic properties. However, advancing Didymin toward clinical application requires further studies on pharmacokinetics, long-term toxicity, and drug-drug interaction synergism. Future research should focus on developing targeted delivery systems and leveraging multi-omics technologies to provide more robust experimental evidence for its potential as a novel LF therapeutic.

Grape Seed Proanthocyanidin (GSP) is a concentrated tannin compound primarily found in *Vitis vinifera L.* *(Vitaceae)*, rich in flavonoid components and exhibiting diverse bioactivities including antioxidant, antibacterial, antiviral, anticancer, and anti-inflammatory effects (Qiao et al., 2023). Lee et al. employed LPS-stimulated human HSCs (LX-2) as a cellular model to investigate GSP’s anti-inflammatory effects and potential molecular mechanisms. Experimental results demonstrated that GSP pretreatment (10, 25 μg/mL) significantly suppressed the mRNA expression of LPS-induced proinflammatory cytokines IL-1β, IL-6, and IL-8, while downregulating mRNA and protein expression levels of iNOS and COX-2. Concurrently, GSP exhibited a dose-dependent inhibition of IκBα phosphorylation and NF-κB p65 activation, while significantly suppressing the phosphorylation and activation of JNK, ERK, p38, and Akt. No cytotoxicity was observed in LX-2 cells within the experimental concentration range (Lee et al., 2017). These findings indicate that GSP exerts anti-inflammatory effects by regulating MAPK, Akt, and NF-κB to suppress the expression of inflammation-related molecules in HSCs, potentially representing a key mechanism underlying its anti-hepatitis and anti-fibrotic properties. However, this study is based solely on *in vitro* cell models and does not involve *in vivo* animal experiments or human clinical trials. Further in-depth research is needed to validate GSP’s anti-inflammatory efficacy, safety, pharmacokinetic characteristics, and specific target mechanisms *in vivo*.

Apigenin (APG), a natural flavonoid compound with multiple biological activities*,* can be extracted from *Matricaria recutita L.* *(Asteraceae)* To investigate its anti-LF mechanism, Qiao et al. established a LF model in Wistar rats induced by CCl_4_ and administered different doses of APG (150, 300, 600 mg/kg) for intervention. Results showed that all APG dose groups improved weight loss and hepatosplenomegaly (with the high-dose group exhibiting optimal effects), reduced hepatic necrosis, inflammatory infiltration, and collagen deposition. Serum liver injury and fibrosis markers (AST, ALT) were reduced, whereas ALB and TP were elevated. APG upregulated the activities of SOD and GSH-PX, decreased MDA levels, ameliorated liver function, mitigated oxidative stress (OS), and suppressed excessive ECM deposition in the liver. qRT-PCR and Western blot validation confirmed that APG significantly downregulated mRNA and protein expression of TGF-β1, α-SMA, and related molecules. Collectively, APG may exert anti-fibrotic effects by regulating multiple signaling pathways through VEGF-mediated FAK phosphorylation, thereby inhibiting HSCs activation and proliferation while promoting apoptosis (Qiao et al., 2020). However, this study has certain limitations: First, the positive control used only the traditional Chinese medicine (TCM) Biejia Ruan Gan Pian, lacking an internationally recognized chemical drug control, which affects the universality of the conclusions. Second, long-term drug safety and toxicological evaluations were not conducted, and pharmacokinetic data support is lacking.

### 5.2 Glycosides

As a major category of plant-derived natural active components, the diverse pharmacological activities of glycosides primarily depend on the chemical structure of the aglycone and the type, number, and linkage pattern of the sugar moieties. Notably, these compounds exhibit multiple properties including anti-inflammatory, antioxidant, anti-liver fibrosis, and lipid metabolism regulation effects, demonstrating promising applications in the prevention and treatment of LF.

Dioscin (DIO), a natural steroidal saponin extracted from medicinal plants such as *Dioscorea nipponica Makino*, *(Dioscoreaceae)* exhibits multiple pharmacological activities including antifungal and antiviral effects (Bandopadhyay et al., 2022). This study investigates its effects and mechanisms on bile duct ligation (BDL)- and dimethylnitrosamine (DMN)-induced LF in rats. *In vivo* experiments demonstrated that continuous administration of DIO at doses of 20, 40 and 60 mg/kg for 4–6 weeks significantly ameliorated weight loss in model rats, reduced serum levels of ALT, AST and TBIL, alleviated hepatic necrosis, inflammatory infiltration and collagen deposition in liver tissue, and downregulated the expression of α-SMA, COL1A1, COL3A1 and fibronectin; these therapeutic effects were superior to those of the positive control drugs UDCA and silymarin. Concurrently, it elevated antioxidant markers such as GSH and SOD while reducing MDA levels, thereby alleviating OS. *In vitro* studies demonstrated that DIO exhibited no significant cytotoxicity at concentrations ≤2.4 μg/mL, inhibited proliferation of rat HSC-T6 and human LX-2 HSCs, reduced ROS production, and suppressed expression of cell activation markers. Mechanistic studies confirm that DIO promotes Nrf2 nuclear translocation, upregulates antioxidant genes such as HO-1 and GST, thereby inhibiting p38 MAPK phosphorylation and reducing synthesis of fibrosis-related proteins. Silencing Sirt1/Nrf2 or inhibiting p38 MAPK with SB-203580 reverses its antifibrotic effects, suggesting dependence on the Sirt1/Nrf2-p38 MAPK (Gu et al., 2016). In summary, DIO exerts multi-targeted anti-fibrotic effects by suppressing OS, blocking hepatic stellate cell activation, and inhibiting collagen deposition, making it a promising therapeutic candidate. Future studies on pharmacokinetics and other aspects are needed to advance its clinical translation.

Astragaloside IV (AS-IV) is a natural saponin compound isolated from the leguminous plants *Astragalus mongholicus Bunge *(*Fabaceae*). Modern pharmacological studies indicate that AS-IV possesses potential preventive and therapeutic effects against LF, primarily by regulating OS-related signaling pathways (Wang M.Y. et al., 2023). In an *in vitro* model of activated HSCs, AS-IV treatment (3, 10, 30, 100 μM) significantly alleviated cellular OS, manifested by scavenging ROS, reducing lipid peroxidation (LPO) levels, and enhancing GSH levels in a dose-dependent manner. Concurrently, AS-IV enhanced cellular antioxidant defense by upregulating Nrf2 gene and protein expression, thereby suppressing α-SMA, Col I, and fibronectin expression and reducing collagen secretion. Mechanistic studies revealed that AS-IV inhibits HSCs activation and excessive ECM production by inducing Nrf2-mediated GSH synthesis and blocking phosphorylation in p38 MAPK. The anti-fibrotic effects of AS-IV were significantly reversed upon pretreatment with BSO or the p38 MAPK-specific inhibitor SB-203580. Furthermore, in regulating fibrosis-related signaling networks, AS-IV showed no significant effect on TGF-β1 secretion, suggesting its antifibrotic action does not depend on TGF-β1/Smad but instead exerts regulatory effects through the specific Nrf2/GSH/p38 MAPK (Li et al., 2013). However, this study was limited to *in vitro* cell experiments and has not been systematically validated in *in vivo* animal models or clinical samples. It lacks comparative efficacy analysis with existing antifibrotic drugs, and its clinical translational value requires further clarification.

Notoginsenoside R1 is an active saponin compound isolated from the roots of *Panax notoginseng (Burk) F. H. Chen* *(Araliaceae)*, exhibiting pharmacological activities including antioxidant, anti-inflammatory, and anti-fibrotic effects (Yang et al., 2025). This study systematically investigated its anti-liver fibrosis potential and underlying mechanisms through *in vivo* experiments. In the CCl_4_-induced rat LF model, intraperitoneal administration of Notoginsenoside R1 (25, 50, 100 mg/kg) for 6 weeks. Notoginsenoside R1 dose-dependently improved histopathological damage in rat liver tissue, elevated ALB and TP levels, and reduced ALP, AST, and ALT activity. Its effects were associated with inhibiting HSCs activation, manifested by downregulating Coll-a1, α-SMA, and TIMP1 expression while upregulating PPAR-γ levels. Concurrently, Notoginsenoside R1 significantly reversed CCl_4_-induced OS (by increasing GSH, SOD, and GST levels while decreasing MDA content) and inflammatory responses (by reducing IL-1β, IL-6, and TNF-α release). This mechanism likely involves inhibiting p-p65, p-ERK, p-JNK, and p-p38 protein phosphorylation in NF-κB and MAPK (Gong et al., 2022). Although this study clarifies that Notoginsenoside R1 exerts anti-liver fibrosis effects through multiple pathways, providing experimental basis for its clinical application, limitations remain. First, the direct molecular targets of this compound remain unidentified, lacking validation experiments such as molecular docking and target enrichment analysis; Second, no comparative efficacy studies with commonly used clinical anti-fibrotic agents were conducted, leaving its clinical translational value to be further substantiated. These issues require further refinement in subsequent research.

Hydroxysafflor Yellow A (HSYA) is an active component of safflower (*Carthamus tinctorius L. *(*Asteraceae*)), a plant belonging to the Asteraceae family. It has been demonstrated to possess pharmacological activities including antifibrotic effects and protection of liver and kidney function (Zhang et al., 2011). Liu et al. employed CCl_4_-combined-with-HFD-induced rat models of LF to investigate the therapeutic potential of HSYA. *In vivo* results showed that HSYA (10 mg/mL) significantly improved liver function indicators in model rats, reduced serum levels of HA, LN, and collagen-related markers, alleviated hepatic inflammatory infiltration and collagen deposition, downregulated expression of pro-fibrotic factors TIMP-1 and TGF-β1, and upregulated expression of PPAR-γ and MMP-2. Mechanistic studies revealed that HSYA exerts its anti-fibrotic effects by activating the PPAR-γ signaling pathway, inhibiting p38 MAPK phosphorylation, and thereby regulating ECM metabolism and inflammatory cytokine release. This effect was reversible upon treatment with the PPAR-γ antagonist GW9662. In summary, HSYA may exert anti-inflammatory and anti-fibrotic effects by regulating PPAR-γ/p38 MAPK, demonstrating significant protective effects against CCl_4_-HFD-induced LF (Liu et al., 2014). However, this study employed only a single HSYA dosage without systematically investigating the dose-response relationship. Furthermore, the direct binding of HSYA to PPAR-γ and its interaction network with downstream signaling molecules remain unclear. The applicability of HSYA across different etiologically induced LF models and its clinical translational value require further validation in subsequent studies.

NPLC0393, a triterpenoid saponin compound isolated from *Gynostemma pentaphyllum (Thunb.) Makino* *(Cucurbitaceae)*, has been demonstrated to possess significant antifibrotic activity. In CCl_4_-induced mouse models of LF, NPLC0393 exerts its antifibrotic effects by modulating the TGF-β1/NDRG2/MAPK signaling axis to inhibit HSCs activation. Specifically, NPLC0393 significantly reversed the CCl_4_-induced downregulation of NDRG2 expression in liver tissue. As a key regulatory molecule, NDRG2 blocks the transdifferentiation of HSCs into myofibroblasts by inhibiting the phosphorylation levels of ERK and JNK. Concurrently, NPLC0393 effectively reduced mRNA and protein expression levels of fibrosis markers α-SMA and COL1A1, diminishing excessive ECM deposition. Mechanistic studies revealed that NPLC0393, by upregulating NDRG2 expression, inhibits TGF-β1-mediated ERK/JNK phosphorylation activation, thereby downregulating transcription of pro-fibrotic genes and ultimately suppressing HSCs activation (Huang et al., 2020). These findings confirm that NPLC0393 exerts potent anti-fibrotic effects by regulating the TGF-β1/NDRG2/MAPK signaling axis, providing novel pharmacological targets and potential therapeutic agents for LF treatment.

### 5.3 Phenolic compounds

Phenolic compounds are a class of natural organic compounds widely distributed throughout the plant kingdom. These compounds exhibit remarkable and diverse biological activities due to their complex molecular structures formed by various functional groups (e.g., hydroxyl, carboxyl, methoxy) attached to aromatic rings and differing degrees of polymerization. These activities include antioxidant effects against stress-induced damage, inhibition of HSCs activation, regulation of fibrosis-related signaling pathways, anti-inflammatory hepatoprotection, anti-apoptotic effects on hepatocytes, and promotion of ECM degradation.

4-Methoxy Sulfonyl Paeonol (4-MSP) is a novel benzenesulfonyl derivative of paeonol, the active component of the traditional Chinese medicine *suffruticosa Andrews* *(Paeoniaceae)*. It has been demonstrated to possess activity inhibiting hepatitis B virus replication. Liao et al. investigated its anti-fibrotic effects and mechanisms using a CCl_4_-induced mouse LF model and *in vitro* experiments with LX2 and HSC-T6 HSCs. Results showed that *in vivo* administration of 5 mg/kg 4-MSP significantly improved CCl_4_-induced liver tissue injury in mice. It downregulated expression of fibrosis markers including α-SMA and col1A2, reduced collagen deposition and hydroxyproline accumulation, and inhibited TNF-α and macrophage marker F4/80 expression without significant toxic side effects.*In vitro* experiments demonstrated that 5 μmol/L 4-MSP dose-dependently inhibited TGF-β1-induced Smad2/3 phosphorylation and downregulated α-SMA and col1A1 protein and mRNA expression. It also blocked PDGF-BB-mediated phosphorylation of MEK/ERK, JNK, p38, and Akt/p70S6K, inhibiting HSCs proliferation and migration without inducing apoptosis. These findings indicate that 4-MSP mitigates LF progression by inhibiting HSCs activation and proliferation, reducing inflammatory cytokine release and collagen deposition through blockade of TGF-β1/Smad, PDGF-BB/MAPK, and Akt (Liao et al., 2020). This study suggests that 4-MSP, as a novel derivative effective at low doses and with a favorable safety profile, holds promise as a potential therapeutic candidate for LF. However, this research did not investigate its long-term toxicity, safety margin, or *in vivo* metabolic mechanisms. These issues require further investigation to advance its clinical application.

Dehydrozingerone (DHZ), a phenolic compound derived from *Zingiber officinale Roscoe* *(Zingiberaceae)*, exhibits anti-inflammatory and antioxidant activities. Sharma et al. investigated its antifibrotic effects and mechanisms using thioacetamide (TAA)-induced rat LF models and a TGF-β-activated human HSCs-LX2 models. *In vivo* experiments demonstrated that oral administration of DHZ (25, 50 mg/kg) for 9 consecutive days significantly improved TAA-induced weight loss and reduced survival rate in rats. It alleviated elevated liver-spleen ratios and hepatic dysfunction, decreased serum AST, ALT, and ALP levels, and dose-dependently inhibited bridging fibrosis, collagen deposition, and pathological deformities in liver tissue. At the molecular level, it downregulated expression of fibrosis markers such as α-SMA and COL1A1, as well as inflammatory factors MPO and NE, while increasing catalase activity to alleviate OS. *In vitro* experiments showed that DHZ (12.5–50 μM) was non-toxic to HSCs-LX2, dose-dependently inhibited TGF-β-induced cell activation, and reduced expression of markers including α-SMA and FN1. Mechanistically, DHZ significantly inhibits phosphorylation of p38, ERK, and JNK both *in vivo* and *in vitro*, thereby blocking HSCs activation and fibrotic signaling (Sharma et al., 2022). In summary, DHZ exerts anti-fibrotic effects by modulating the MAPK Signaling Pathway to inhibit hepatic stellate cell activation, mitigate OS and inflammatory responses, and reduce expression of fibrosis markers and collagen deposition. This provides experimental evidence for developing anti-fibrotic drugs from natural drugs and positions DHZ as a potential therapeutic candidate for LF.

Chlorogenic acid (CGA), a phenolic compound widely present in *Lonicera japonica Thunb.*, *(Caprifoliaceae)* exhibits multiple bioactivities including anti-inflammatory and antioxidant effects. Its potential in LF treatment has garnered significant attention in recent years. Shi et al. investigated the intervention effects and molecular mechanisms of CGA on CCl_4_-induced LF through *in vivo* and *in vitro* experimental systems. *In vivo* experiments demonstrated that oral administration of CGA (60 mg/kg) to CCl_4_-induced fibrotic rats significantly reduced fibrosis severity and hydroxyproline content, improved lobular structure disruption and excessive collagen deposition, and downregulated α-SMA, type I collagen, type III collagen, and TIMP-1 expression. Concurrently, CGA decreased MDA levels in liver tissue, elevated GSH content and SOD/CAT activity, inhibited CYP2E1 expression, and promoted Nrf2 nuclear translocation. *In vitro* experiments stimulated rat hepatic stellate cell line HSC-T6 with PDGF revealed that CGA (12.5, 25, 50 μg/mL) concentration-dependently inhibited p47phox and gp91phox expression, reduced ROS generation, suppressed p38 and ERK1/2 phosphorylation, and consequently inhibited HSCs proliferation and fibrosis-related gene expression (Shi et al., 2016). This indicates that CGA exerts anti-fibrotic effects by activating the Nrf2 antioxidant pathway and inhibiting PDGF-mediated NOX/ROS/MAPK. However, its direct targets and upstream/downstream regulatory relationships within these pathways remain unclear. Given potential crosstalk between Nrf2 and NOX/ROS/MAPK, future studies should elucidate core regulatory mechanisms.

Curcumin, a natural polyphenolic compound extracted from *Curcuma longa L.* *(Zingiberaceae)*, exhibits significant pharmacological activities including antifibrotic and anti-inflammatory effects (Gorabi et al., 2020). Hu et al. investigated its mechanisms using a TAA-induced mouse LF model and *in vitro* HSCs culture. *In vivo*, 4 weeks of curcumin (400 mg/kg) intervention significantly reduced LF severity and decreased MAT2B expression in activated HSCs. *In vitro*, 24-h treatment of HSCs with 20 μM curcumin downregulated MAT2B expression at both mRNA and protein levels. Mechanistic studies revealed that curcumin blocks p38 MAPK phosphorylation activation, thereby inhibiting its mediation of MAT2B promoter activity upregulation. This reduces MAT2B transcription and translation, decreases collagen α1 mRNA expression, and upregulates antioxidant pathway molecules to mitigate OS damage. AAV-mediated MAT2B gene silencing experiments confirmed that MAT2B downregulation is a key component of curcumin’s inhibition of HSCs activation and LF (Hu and Zhou, 2020). In summary, curcumin modulates HSCs activation and inhibits collagen deposition by targeting the p38 MAPK/MAT2B signaling axis, thereby improving the progression of LF. Its multi-level regulatory mechanism provides robust experimental evidence for its development and clinical application as a natural anti-fibrotic drug.

### 5.4 Terpenoids

Terepenoid compounds represent a diverse class of naturally occurring organic molecules widely distributed in nature. They are present in plants, microorganisms, and certain animals, particularly concentrated in plant secretions such as essential oils, resins, and pigments, as well as in the active components of medicinal fungi. Leveraging diverse molecular skeletons and functional group structures based on the isoprene unit, these compounds exhibit significant and targeted anti-fibrotic activity. They exert multiple important pharmacological effects, including blocking pro-fibrotic signaling pathways and promoting apoptosis in fibrosis-related cells.

Carvacrol is a monoterpenoid compound widely distributed in *Origanum vulgare L.* *(Lamiaceae)*, exhibiting diverse pharmacological activities including antifungal, antitumor, and anti-inflammatory effects (Sharifi-Rad et al., 2018). Cai et al. elucidated the anti-hepatic fibrosis effects and associated molecular mechanisms of carvacrol utilizing CCl_4_-induced murine LF models and PDGF-BB-activated rat HSC-T6 hepatic stellate cells. Results demonstrated that *in vivo*, continuous 6-week oral administration of carvacrol at different concentrations (25, 50, 100 mg/kg) dose-dependently improved CCl_4_-induced hepatic histopathological damage in mice. It reduced liver-to-body weight ratio, decreased levels of liver injury markers such as ALT and AST, and suppressed expression of fibrosis indicators including α-SMA and Col1α1. *In vitro* experiments demonstrated that carvacrol effectively inhibited PDGF-BB-induced proliferation and activation of HSC-T6, reducing collagen production. Mechanistically, carvacrol exerts its effects by inhibiting the phosphorylation levels of ERK1/2, JNK1/2, and p38 MAPK. However, this study did not investigate the long-term toxicity or pharmacokinetics of carvacrol, leaving a lack of data to support its safety and dose optimization for clinical translation (Cai et al., 2021). In summary, this study preliminarily confirms carvacrol’s core role in alleviating LF by inhibiting HSCs activation and proliferation and regulating MAPK. However, the aforementioned limitations constrain the depth of the conclusions and their clinical translational value. Targeted mechanism validation and safety assessment studies are required for future research.

Betulinic acid (BA), a natural triterpenoid compound isolated from *Ziziphus jujuba Mill.* (*Rhamnaceae*), exhibits anti-inflammatory, antitumor, and hepatoprotective potential. This study investigated its effects and mechanisms in human HSCs *in vitro* and in CCl_4_-induced mouse LF models *in vivo*. Groups included a control group, model group, colchicine positive control group (0.2 mg/kg), and BA low-, medium-, and high-dose groups (15, 30, 60 mg/kg), with 6 weeks of intervention. Results showed that CCl_4_-induced mice exhibited marked hepatic injury, elevated ALT, AST, Hypoxanthine (Hyp), and PDGF-BB levels, along with hepatic hemorrhage, necrosis, increased collagen deposition, and upregulation of α-SMA and COL-1 expression. BA significantly ameliorated these injuries across all dose groups, with particularly pronounced effects in the medium and high-dose groups. It also exhibited a dose-dependent upregulation of LC3B-II and ATG7 gene and protein expression, promoting the autophagic flux. *In vitro* experiments demonstrated that BA increased the number of autophagosomes in HSCs and reduced α-SMA expression. However, its anti-fibrotic effect was attenuated when combined with autophagy inhibitors. Mechanistically, BA had no significant effect on the PI3K/AKT/mTOR/p70S6K or STAT3 pathways but markedly inhibited phosphorylation of key proteins in the MAPK/ERK pathway (Liu N. et al., 2019). This study first demonstrates that BA exerts anti-fibrotic effects by inducing autophagy and downregulating α-SMA through MAPK/ERK, providing a novel natural candidate drug for therapy. However, the study did not explore the combined efficacy of BA with clinical anti-fibrotic drugs nor clarify its long-term safety, warranting further investigation.

Oridonin, the core active diterpene component of *Rabdosia rubescens (Hemsl.) Hara* *(Lamiaceae)*, has been demonstrated to possess diverse pharmacological activities. Using HSC-T6 as models, this study investigated its anti-LF potential. Results showed oridonin concentration-dependently inhibited cell viability. It induced morphological changes such as vesicle formation and apoptotic body generation, increased the proportion of subG1 phase cells and DNA fragmentation levels, activated Caspase 3, reduced mitochondrial membrane potential, and ultimately triggered apoptosis. At the mechanistic level, oridonin concentration- and time-dependently depleted intracellular GSH and promoted ROS generation. NAC and GSH-MEE reversed these effects, confirming the central role of OS in apoptosis. Additionally, oridonin activates ERK, JNK, and p38 MAPK phosphorylation, yet specific inhibitors fail to block apoptosis, suggesting that pathway activation depends on GSH depletion-mediated OS but does not directly regulate apoptosis (Kuo et al., 2014). However, this study has certain limitations: it employed only an *in vitro* HSCs-T6 model, lacking *in vivo* animal experiments to validate Oridonin’s antifibrotic effects; it did not investigate the differential actions of different Oridonin concentrations in normal hepatocytes versus HSCs, making it difficult to assess its clinical safety. Future work should involve *in vivo* experiments and safety evaluations to further refine the mechanism of Oridonin’s antifibrotic action in the liver, providing more robust experimental evidence for its clinical translation.

Tanshinone IIA (Tan IIA), the primary active component of *Salvia miltiorrhiza Bunge* *(Lamiaceae)* ethanol extract, is a diterpenoid quinone derivative exhibiting significant anti-fibrotic and apoptosis-inducing pharmacological activities (Shan et al., 2025). Research by Pan et al. confirmed that Tan IIA exhibits a dose-dependent inhibition of proliferative activity in the rat hepatic stellate cell line HSC-T6. Furthermore, Tan IIA effectively ameliorates DMN-induced hepatic fibrosis pathology in rats by reducing collagen deposition and α-SMA expression, exerting its antifibrotic effects through inducing apoptosis in activated HSCs. Mechanistic studies indicate Tan IIA significantly upregulates prohibitin protein expression, promotes cytoplasmic C-Raf translocation to the membrane for complex formation, thereby activating MAPK and inhibiting AKT phosphorylation. By enhancing ERK phosphorylation, Tan IIA upregulates Bax expression, suppresses Bcl-2, promotes cytochrome c release from mitochondria into the cytoplasm, activates the Caspase-3/Caspase-9 cascade, and induces PARP cleavage, ultimately inducing HSCs apoptosis (Pan and Wang, 2012). Although this study elucidates Tan IIA’s anti-fibrotic mechanism via the C-Raf/prohibitin-mediated ERK-Bax-caspase pathway, the experiments primarily relied on cell lines and rat models. The absence of organoid or humanized model validation, along with preclinical pharmacokinetic assessments, limits the exploration of its specificity and clinical translation potential. These limitations constrain the extrapolation of this compound’s clinical applicability.

Curcumol, a natural active component derived from the traditional Chinese medicine *Curcuma phaeocaulis Valeton (Zingiberaceae)*, exhibits diverse pharmacological activities including anti-inflammatory and anti-tumor effects. In recent years, it has garnered significant attention in the field of anti-liver fibrosis (Zheng et al., 2022). This study demonstrated curcumol’s therapeutic efficacy in CCl_4_-induced mouse LF models through *in vivo* and *in vitro* experiments. Gao et al. found that Curcumol significantly inhibited LSEC migration and luminal formation in a dose-dependent manner, reduced Angiopoietin 2 and VEGFR-2 expression, alleviated inflammatory infiltration and collagen deposition in mouse liver tissue, and improved serum ALT, AST, and LF markers HA and LN levels. Mechanistic studies indicate that Curcumol exerts anti-angiogenic effects by inhibiting transcription factor KLF5 expression, with KLF5 overexpression reversing its therapeutic effects. Further research confirms Curcumol reduces mitochondrial ROS production in LSECs, improves mitochondrial morphology and membrane potential, and downregulates KLF5 expression by blocking the ROS/ERK signaling pathway, thereby inhibiting LSEC-mediated pathological angiogenesis. Additionally, Curcumol upregulates SOD activity and GSH/GSSG ratio while reducing MDA levels, enhancing cellular antioxidant stress resistance (Gao et al., 2021). This study has limitations: the experimental model exclusively utilized CCl_4_-induced mouse LF, lacking validation in other etiological models; preclinical pharmacokinetic and toxicological studies were not conducted, leaving Curcumol’s long-term safety, *in vivo* metabolic characteristics, and optimal dosage undefined. Future research should incorporate diverse etiological LF models and human cell experiments, complementing pharmaceutical data to provide more robust theoretical and experimental support for clinical translation.

Mogroside IVE (MGIVE) is a major natural cucurbitane-type triterpenoid compound isolated from *Siraitia grosvenorii (Swingle) C.* *(Cucurbitaceae)* It exhibits broad pharmacological activity with extremely low toxicity, demonstrating potential therapeutic value in LF treatment (Wang L. et al., 2023). Cao et al. comprehensively evaluated MGIVE’s antifibrotic effects and molecular mechanisms using CCl_4_-induced mouse LF models, TGF-β1- or LPS-stimulated HSCs (HSC-T6), and the RAW264.7 model. *In vitro* experiments demonstrated that 10 μM MGIVE significantly inhibited TGF-β1- or LPS-induced HSCs activation, downregulated α-SMA, Col I, and HIF-1α expression, modulated MMP-1/TIMP-1 balance, and reduced inflammatory cytokine levels. *In vivo* studies demonstrated that MGIVE (25 mg/kg) administered via oral gavage significantly ameliorated CCl_4_-induced liver injury in mice, reducing ALT and AST activity while mitigating hepatic inflammatory infiltration and collagen deposition. This effect was mediated through inhibition of the TLR4/MyD88 pathway and MAPK phosphorylation (Cao et al., 2018). These studies indicate that MGIVE inhibits HSCs activation and inflammatory responses by regulating the TLR4/HIF-1α signaling pathway, offering a novel therapeutic candidate for LF. However, this study has certain limitations: it employed only a single CCl_4_-induced acute LF model and did not thoroughly investigate MGIVE’s metabolic conversion and targeted delivery efficiency *in vivo*. These factors may limit its applicability for clinical translation, necessitating further research to address these gaps.

### 5.5 Lignans

Predominantly found in plant tissues such as xylem, bark, leaves, and fruits. They are particularly concentrated in the active parts of medicinal plants with dual food and medicinal properties, including *Schisandra chinensis (Turcz.) Baill.*, *Forsythia suspensa (Thunb.) Vahl*, and *Eucommia ulmoides Oliv*. These compounds exhibit distinct and multi-targeted anti-liver fibrosis activity due to their diverse molecular configurations and active functional group structures derived from the polymerization of two phenylpropanoid derivatives. They possess multiple key pharmacological effects, including inhibiting the activation and proliferation of HSCs, scavenging OS damage, regulating the cytokine network associated with LF, and repairing damaged liver tissue.

Schizandrin *C*, a lignan compound derived from *Schisandra chinensis (Turcz.) Baill. (Schisandraceae)*, exhibits antioxidant and anti-hepatotoxic properties. Chen et al. investigated its therapeutic potential in CCl_4_-induced mouse models of LF. The results showed that oral administration of Schizandrin C (50 mg/kg) for 4 consecutive weeks significantly improved serum biochemical indicators (decreased ALT, AST, and TBIL activity), reduced hydroxyproline content and collagen deposition, improved liver tissue structure, and downregulated α-SMA and type I collagen expression. *In vitro*, it inhibited activation of LX-2 and HSC-T6 HSCs (Chen J. et al., 2023). Mechanistically, it improves lipid metabolism disorders by regulating 36 differentially expressed lipid molecules, downregulates mRNA levels of inflammatory factors such as IL-6 and TNF-α, and inhibits phosphorylation of IKKβ and p65 subunits in NF-κB, as well as phosphorylation in ERK/p38 MAPK. Research confirms that Schizandrin C exerts lipid metabolism regulation, anti-inflammatory effects, and anti-hepatocyte activation by synergistically modulating these two pathways, thereby improving LF. Limitations of this study include single-dose intervention without establishing dose-response relationships, lack of long-term safety assessment and toxicity analysis, and insufficient exploration of the interactive regulatory mechanisms between lipid metabolism and inflammatory responses.

Schisantherin A (SCA), an active lignan monomer *from Schisandra chinensis (Turcz.) Baill.* *(Schisandraceae)*, significantly ameliorates TAA-induced LF in mice. *In vivo* studies by Wang et al. revealed that SCA (1, 2, 4 mg/kg) alleviated weight gain retardation, elevated liver index, and increased ALT/AST activity in fibrotic mice. It improved hepatic histopathological disarray and collagen deposition while reducing α-SMA, COL1A1, and Hypoxanthine (Hyp) levels in liver tissue and HSC-T6 cells, demonstrating a dose-dependent antifibrotic effect. Mechanistically, SCA maintains hepatic microenvironment stability by upregulating tight junction-associated proteins and inhibits TGF-β1-mediated TAK1/MAPK (p-TAK1, p-JNK, p-p38) and NF-κB (p-p65, p-IκBα) pathways, thereby reducing the release of inflammatory mediators such as TNF-α, IL-1β, and IL-6. This ultimately inhibits HSCs activation and inflammatory cascades. *In vitro* experiments confirmed that SCA (0.625–5 μM) inhibited TGF-β1-induced HSC-T6 proliferation and reduced inflammatory cytokine levels in LPS-stimulated RAW264.7 cells, corroborating *in vivo* findings. This study elucidates SCA’s anti-fibrotic mechanism through TGF-β1/TAK1/MAPK/NF-κB pathway regulation and HSCs activation inhibition, providing experimental basis for developing Schisandra-derived natural anti-fibrotic drugs (Wang H. et al., 2021). However, the study has limitations: it did not conduct pharmacokinetic studies, liver-targeted distribution analysis, or long-term toxicity assessments, making it difficult to support feasibility analysis for clinical translation. Furthermore, the *in vitro* experiments used only the HSC-T6 cell line without validation in primary cells, which may affect the clinical relevance of the results and requires further refinement.

### 5.6 Alkaloid compounds

Alkaloids are nitrogen-containing basic organic compounds (often heterocyclic structures) predominantly found in plant tissues such as roots, stems, leaves, fruits, and seeds. These compounds exhibit distinct, multi-targeted anti-fibrotic activity against LF due to their nitrogen-containing heterocyclic core structures and diverse substituent modifications. They exert multiple key pharmacological effects, including inhibiting HSCs transdifferentiation and proliferation, reducing inflammatory infiltration in liver tissue, improving hepatic microcirculation, and repairing damaged hepatocytes.

Neferine, a dibenzylisoquinoline alkaloid extracted from the seed embryo of *Nelumbo nucifera Gaertn.* (*lotus*) *(Nelumbonaceae)*, has been demonstrated to possess multiple pharmacological activities including anti-inflammatory, antioxidant, and anti-fibrotic effects (Wang L. et al., 2023). Wang et al. Employed CCl_4_-induced rat LF models to investigate Neferine’s therapeutic potential. *In vivo* results showed that Neferine (5, 10 mg/kg) significantly improved weight loss in CCl_4_-induced rats, reduced liver index, improved liver function markers (ALT, AST, TBIL) and fibrosis markers (HA, PIIINP, IV-C, LN) levels, decreased inflammatory infiltration and collagen deposition in liver tissue, and upregulated antioxidant enzyme activities SOD, GSH-PX, and CAT while decreasing MDA levels. Mechanistic studies revealed that Neferine inhibits phosphorylation of p38 MAPK, ERK1/2, and JNK, concurrently preventing IκBα degradation and NF-κB p65 nuclear translocation, thereby downregulating expression of inflammatory factors including α-SMA, TGF-β1, TNF-α, and IL-6 (Wang Y. et al., 2021). These findings confirm that Neferine may exert antioxidant and anti-inflammatory effects by inhibiting MAPK and NF-κB/IκBα, thereby conferring protective effects against CCl_4_-induced LF. It is noteworthy that although this study included different dosage groups, it did not conduct an in-depth analysis of the dose-response characteristics. Furthermore, the dose dependency of some indicators remains unclear. The applicable dosage for clinical translation and the differences in efficacy for LF with various etiologies require further validation in subsequent studies.

### 5.7 Other compounds

Ophiopogonis Radix Inulin New Series Fructan (OJP-W2) is a homogeneous fructan purified from the dried rhizomes of *Ophiopogon japonicus (L. f) Ker-Gawl*. *(Asparagaceae)*, a plant belonging to the genus Ophiopogon in the family Liliaceae. Liu et al. Established mouse LF models via intraperitoneal injection of a 15% CCl_4_ olive oil solution to demonstrate OJP-W2’s therapeutic potential. *In vivo* experiments demonstrated that both OJP-W2 (50, 100 mg/kg) and sorafenib (5 mg/kg) significantly improved liver swelling and elevated liver-body ratio in model mice, mitigated hepatocyte injury and collagen deposition, reduced serum ALT and AST levels, decreased liver tissue hyaluronic acid (Hyp) content, and downregulated fibrosis markers including HA and collagen IV. Mechanistic investigations demonstrated that OJP-W2 exerts its biological effects through multiple pathways: it inhibits the activation of the TGF-β/Smads signaling pathway, thereby downregulating the expression of α-SMA and COL1A1; suppresses the MAPK pathway by reducing the phosphorylation of p38, ERK and JNK, which in turn attenuates the release of inflammatory factors including IL-1β and IL-6; and modulates the Bax/Bcl-2 balance to inhibit ATF4/Caspase 3-mediated hepatocyte apoptosis (Liu et al., 2023). In summary, OJP-W2 exerts anti-fibrotic effects by regulating fibrosis-, inflammation-, and apoptosis-related signaling pathways, enriching the typology and pharmacological activity research of Ophiopogon fruit polysaccharides. However, this study did not explore the structure-activity relationship of OJP-W2 in depth, nor did it clarify the molecular mechanisms underlying the dose-dependent effects. Its pharmacokinetic characteristics and long-term safety in clinical translation require further validation.

Salvianolic acid B (Sal B) is the primary active component among water-soluble phenolic acids in *Salvia miltiorrhiza Bunge* *(Lamiaceae)*, exhibiting diverse pharmacological activities including antioxidant, anti-inflammatory, and anti-fibrotic effects (Fu et al., 2024). *In vivo* and *in vitro* experiments by Wu et al. demonstrated that Sal B (*in vivo*: 15, 30 mg/kg; *in vitro* 25, 50, 100 μM) significantly improved pathological features of diethylnitrosamine (DEN)-induced LF in mice, reduced levels of fibrosis markers including α-SMA, Collagen I, and TGF-β, and simultaneously inhibited TGF-β-activated HSCs (HSC-T6, LX-2) in terms of activation, collagen production, and migration capacity. Mechanistically, Sal B inhibited ERK, JNK, and p38 phosphorylation, downregulates phosphorylation of the Smad2/3 linker region (P-Smad2L/3L) and Smad2 C-terminal region (P-Smad2C), upregulates Smad3 C-terminal phosphorylation (P-Smad3C), and inhibits downstream PAI-1 expression. This suggests its action is mediated through regulation of the TGF-β/MAPK/Smad pathway (Wu C. et al., 2019). These studies clarify that Sal B exerts its anti-fibrotic effects by modulating MAPK-mediated Smad2/3 phosphorylation isoform signaling, providing a theoretical basis for its clinical application. However, studies have not conducted site-specific mutation experiments on the linker region of Smad2/3 phosphorylation, nor have they been validated in LF models induced by different etiologies. This prevents the complete exclusion of synergistic effects from other signaling pathways, and the specificity and universality of the mechanism still require further confirmation.

### 5.8 Extracts

Extracts are complex bioactive substances derived from natural drugs (plants, animals, microorganisms, etc.), often containing polyphenols, flavonoids, terpenoids, and other components. They are typically found in specific tissues, secretions, or metabolites of natural sources. These substances exhibit distinct and multi-pathway anti-fibrotic activity through the synergistic effects of their diverse bioactive components and rich structural diversity. They possess multiple key pharmacological actions, including inhibiting HSCs activation and collagen deposition, as well as alleviating inflammatory responses and OS in liver tissue.

Total polyphenolic glycoside extract (TPLR) is an active fraction isolated from Tibetan medicinal plants *Phlomoides rotata (Benth. ex Hook.f.) Mathiesen* *(Lamiaceae)*, rich in phenylpropanoid and flavonoid compounds, and has demonstrated anti-LF activity. Yang et al. demonstrated that TPLR (25–50 μg/mL) significantly reduced TGF-β-induced α-SMA and Desmin protein expression in LX-2 cells, upregulated quiescence-related transcription factors such as ETS1 and GATA4, downregulated activation-related factors like AP1 and NF-κB, and induced the transformation of activated HSCs toward a quiescent-like phenotype. *In vivo* with CCl_4_-induced LF mouse models, TPLR (0.05–0.2 g/kg) mitigated hepatic tissue injury and collagen deposition while reducing ALT, AST, TNF-α, and IL-6 levels. The high-dose group exhibited efficacy comparable to positive controls colchicine (0.2 mg/kg) and Fuzheng Huayu Capsules (4.6 g/kg). Mechanistically, transcriptomic and proteomic analyses confirmed the AGE/RAGE signaling pathway as a core target. TPLR exerts its antifibrotic effects by downregulating RAGE and its downstream RAS/MAPK/NF-κB axis activity while inhibiting AGE formation (Yang et al., 2024). However, this study has limitations: *In vitro* studies did not clarify the direct inhibitory potency of TPLR on AGEs or establish the correlation between *in vitro* and *in vivo* effects; the contribution of the 14 major components’ activities and monomer-level target validation remain unconfirmed; interactions between the microenvironment (e.g., gut microbiota) and the AGE/RAGE pathway were not thoroughly explored. In summary, TPLR may exert its antifibrotic effects by regulating hepatic stellate cell phenotypic transformation mediated through AGE/RAGE. Further component characterization and multidimensional mechanism validation are required to solidify its clinical application foundation.

Lamiophlomis Herba (LH) is an extract derived from *Phlomoides rotata (Benth. ex Hook.f.) Mathiesen* *(Lamiaceae)*, a precious medicinal plant endemic to the Qinghai-Tibet Plateau. Rich in components such as cycloartenolides, phenethyl glycosides, and flavonoids, it has garnered significant attention for its pharmacological activities including promoting blood circulation, dispersing blood stasis, and exhibiting anti-inflammatory and antibacterial effects. Chen et al. found that the extract of LH and its major active components, including acteoside, 8-O-acetylshanzhiside methyl ester (8-O-ASME), luteolin and shanzhiside methyl ester (SME), could effectively alleviate LF induced by HSC-T6 cells. This is evidenced by reduced levels of fibrosis markers such as α-SMA, PC-III, Col-IV, and LN, along with reduced deposition of ECM components like fibronectin, Col1a1, and Col3a1. Additionally, LH and its active components significantly suppressed the expression of pro-inflammatory genes such as PTGS2, EGFR, and SRC, regulated the activity of signaling molecules like AKT1 and MAPK, and improved the imbalance between OS and inflammatory responses. Mechanistically, LH may exert its anti-fibrotic effects by targeting signaling pathways such as MAPK and EGFR/SRC to inhibit HSCs activation and reduce excessive ECM secretion. This study systematically screened active components and potential targets using UPLC-Q-TOF-MS combined with network pharmacology and molecular docking techniques, providing scientific evidence for the pharmacodynamic basis of LH (Chen J. et al., 2023). However, the study has limitations: *in vitro* cell models cannot fully replicate the complex *in vivo* pathological microenvironment, and dose-response analysis across different gradients is lacking. The applicability of LH across various etiologies of LF and its long-term safety require further validation. These findings suggest that attention should be paid to the *in vivo* metabolic transformation of LH’s active components and their interactions with the gut microbiota, providing more comprehensive experimental support for its clinical intervention in LF.

Ginkgo biloba extract (GBE) is a natural phytochemical derived from *Ginkgo biloba L.* *(Ginkgoaceae)*, containing active components such as terpenoid lactones and flavonoids. It has been demonstrated to possess multiple pharmacological activities, including hepatoprotective effects. *In vivo* experiments demonstrated that GBE (15, 30 mg/kg) effectively mitigated weight loss, improved liver function, reduced fibrosis indices, and alleviated histopathological damage in CCl_4_-induced fibrotic rats, with higher doses yielding more pronounced effects. *In vitro* mechanism studies indicate that GBE dose-dependently inhibits p38 MAPK phosphorylation and IκBα degradation, thereby downregulating NF-κB p65 nuclear translocation. Concurrently, it upregulates Bcl-2 expression, downregulates Bax and Caspase-3 activation, and suppresses the expression of hepatic stellate cell activation markers and inflammatory cytokines (Wang et al., 2015). These studies indicate that GBE exerts a multi-target synergistic effect by regulating p38 MAPK, NF-κB/IκBα, and Bcl-2/Bax signaling pathways to inhibit HSCs activation, mitigate inflammatory responses, and reduce hepatocyte apoptosis, providing a novel mechanistic explanation for its therapeutic potential in LF. However, this study has certain limitations: the specific active components in GBE playing a core role remain unidentified, and validation is limited to animal models, lacking clinical data to support its efficacy and safety in humans. Additionally, the interaction mechanism between p38 MAPK and NF-κB/IκBα has not been sufficiently explored. Future research is needed to advance its clinical translation.

Cichorium pumilum Jacq ethyl acetate extract (CGEA) is a natural product derived from the dried roots of the Uyghur traditional medicinal herb *Cichorium pumilum Jacq* *(Asteraceae)*. It primarily contains sesquiterpene compounds and exhibits pharmacological activities such as anti-inflammatory and hepatoprotective effects. Han et al. demonstrated the efficacy and potential mechanisms of CGEA in ameliorating TNBS-induced colitis-associated LF. CGEA alleviates hepatic congestion, tissue damage, and colonic ulceration in fibrotic rats, reduces serum AST and γ-GT levels along with hepatic IL-6 content, increases the Firmicutes/Bacteroidetes ratio in the gut, promotes proliferation of Bifidobacterium adolescentis and Ruminococcus, and restores intestinal barrier function. Its major component, lactucin, inhibits p38, ERK, and AKT protein phosphorylation in LPS-activated RAW264.7 cells, downregulates iNOS and COX-2 expression, and reduces IL-6 and NO release (Han et al., 2021). In summary, CGEA improves the “gut-liver axis” cycle by regulating gut microbiota and repairing the intestinal barrier, while exerting anti-inflammatory effects through the MAPK/AKT, thereby alleviating LF. However, while this study confirmed the involvement of the MAPK/AKT, it did not elucidate the key molecular targets of lactucin’s action. Furthermore, it failed to investigate the causal relationship between gut microbiota changes and signaling pathway activation, lacking direct validation of the specific molecular mechanisms regulating the “gut-liver axis.” These limitations restrict the completeness of its pharmacological mechanism and clinical translational value.

Sasa extract (SE) is a natural bioactive compound derived from *Sasa veitchii (Carrière) Rehder* *(Poaceae)*, a grass species, exhibiting antioxidant, anti-inflammatory, and antitumor properties. Yoshioka et al. conducted *in vivo* experiments using CCl_4_-induced mouse models of LF, administering SE (0.1 mL/day) for 8 weeks with olive oil and saline as controls. Results demonstrated that SE significantly improved CCl_4_-induced weight loss in mice, suppressed elevated liver-to-body weight ratios, reduced plasma levels of ALT, AST, and the inflammatory factor TNF-α, while decreasing MDA production in liver tissue and restoring GSH content. Histopathological analysis indicated that SE mitigated hepatic necrosis and collagen deposition and inhibited α-SMA expression. Mechanistic studies confirmed that SE blocks OS and inflammation-mediated HSCs activation by inhibiting NF-κB p65 nuclear translocation and phosphorylation of p38 MAPK, ERK1/2, JNK (Yoshioka et al., 2018). This study provides robust *in vivo* evidence for SE’s potential in preventing and treating LF. However, significant limitations exist: it employed only a single mouse strain and a chemically induced model without *in vitro* validation; the core active component responsible for its effects remains unidentified; and long-term toxicity assessments and dose-dependent studies are lacking. These shortcomings constrain its translation from basic research to clinical application.

Pomegranate peel extract (EPP), a natural extract rich in polyphenols and flavonoids derived from *Punica granatum L.* *(Lythraceae)*, exhibits diverse pharmacological activities including antioxidant, anti-inflammatory, and anti-fibrotic effects. Experimental results by Wei et al. indicate that EPP (150 mg/kg) significantly improves liver function in CCl_4_-induced fibrotic rats, reduces serum ALT, AST, and T-Bil levels, decreases MDA content in liver tissue, enhances GSH-Px activity, and simultaneously inhibits TGF-β, collagen1-α2, and α-SMA expression, thereby reducing the area of LF. Mechanistically, EPP inhibits p38 MAPK phosphorylation and upregulates Nrf2 along with its downstream targets HO-1 and Nqo1, suggesting its protective effects may be mediated through p38 MAPK/Nrf2 (Wei et al., 2020). These findings demonstrate that EPP exerts significant protective effects in CCl_4_-induced LF by suppressing OS, reducing collagen deposition, and modulating relevant signaling pathways. Although this study clarified the expression changes of key molecules in the p38 MAPK/Nrf2 pathway, providing theoretical support for EPP’s intervention mechanism, it did not directly validate the molecular interaction between p38 MAPK and Nrf2. Furthermore, the lack of long-term safety assessment data limits the confirmation of its clinical translational value in LF treatment.

## 6 Role of natural formulas in liver fibrosis treatment

Natural formulas are complex preparations composed of multiple herbal ingredients following the principles of sovereign, ministerial, assistant, and messenger herbs (primarily in the form of compound decoctions, pills, etc.), often derived from classical formulas and clinical experience. These formulations exhibit distinct and moderate anti-fibrotic activity through their multi-component, multi-target synergistic effects. Key pharmacological actions include inhibiting HSCs activation and collagen deposition, alleviating chronic inflammatory responses in liver tissue, and promoting liver tissue repair and regeneration.

Tianhuang Formula (THF), derived from Professor Guo Jiao’s theory of “regulating the liver, activating the pivot, and transforming turbidity,” is a traditional Chinese medicine formula comprising *Panax notoginseng (Burkill) F.H.Chen (Araliaceae)* and *Coptis chinensis Franch (Ranunculaceae)*. It has demonstrated significant therapeutic effects in metabolic liver diseases. This study investigated the intervention effects and mechanisms of THF using two mouse models of LF induced by CCl_4_ and MCD diet, with Fuzheng Huayu Capsules (FZHY) as the positive control. THF (80, 160 mg/kg) was administered for 4–6 weeks. Results showed that THF significantly alleviate serum ALT, AST, and hepatic TG levels in model mice, while suppressing expression of fibrosis markers α-SMA, Collagen-I, TNF-α, and IL-1β. Higher doses yielded superior effects comparable to FZHY. Transcriptome analysis and validation experiments confirmed that THF downregulates the expression of the macrophage marker CD68. By inhibiting the CCL2-CCR2 axis and its downstream MAPK/NF-κB Signaling Pathway, THF reduces inflammatory cell infiltration and HSCs activation, thereby alleviating liver tissue inflammation and fibrosis (Lan et al., 2024). This study provides experimental support for THF in treating LF, though limitations exist: Only two animal models were used, lacking validation with clinical samples; the active components and dose-response relationship through which THF exerts its core effects remain unidentified; molecular details of the cross-regulation between the CCL2-CCR2 axis and MAPK/NF-κB pathways are insufficiently elucidated, with no *in vitro* experiments to support these findings; and the direct targets and synergistic mechanisms of the compound formulation’s components remain unexplained. Future clinical studies are required to identify key bioactive components through multi-technique screening, elucidate pathway interaction mechanisms, and strengthen the scientific evidence chain to advance clinical translation.

Yu Jin Pulvis (YJP) is a traditional Chinese herbal formula originating from Yanbian, China. It comprises *Curcuma aromatica Salisb. (Zingiberaceae)*, *Boswellia sacra Flück. (Burseraceae)*, *Taraxacum mongolicum Hand.-Mazz. (Asteraceae)*, *Typha angustifolia L. (Typhaceae)*, *Vigna radiata (L.) (Fabaceae)R.Wilczek (Araliaceae)*, *Panax notoginseng (Burk.(Burseraceae)) F.H.Chen*, *Commiphora myrrha (T.Nees) Engl*. It possesses effects such as clearing liver fire, promoting bile secretion, clearing heat, activating blood circulation, resolving stasis, and detoxification. It is widely used in the clinical treatment of patients with liver cirrhosis, fatty liver, and chronic hepatitis. To investigate its anti-liver fibrosis mechanism, Wu et al. Established CCl_4_-induced mouse LF models. YJP (100, 200, 300 mg/kg) was administered via oral gavage for 6 weeks, with silymarin (100 mg/kg) and Fuzheng Huayu Capsules (2 g/kg) serving as positive controls. Results demonstrated that all YJP dose groups significantly ameliorated CCl_4_-induced liver tissue damage in mice, reduced collagen deposition, lowered serum ALT and AST levels, and decreased expression of proinflammatory factors such as TNF-α and IL-1β. Moreover, YJP exhibited a dose-dependent inhibition of α-SMA and Col1 mRNA and protein expression. Further mechanistic investigations demonstrated that YJP markedly downregulated the expression of phosphorylated extracellular signal-regulated kinases (p-ERK, p-JNK, p-p38 MAPK) in the MAPK Signaling Pathway, as well as phosphorylated PI3K (p-PI3K) and phosphorylated Akt (p-Akt) in the PI3K/Akt pathway (Wu W. et al., 2019). This study has significant limitations: the absence of *in vitro* experimental validation, the failure to identify the core monomeric component responsible for YJP’s anti-liver fibrosis effects, and the inability to precisely elucidate its direct molecular targets in regulating signaling pathways all constrain the compound’s clinical translation. The cross-regulatory relationship between the MAPK and PI3K/Akt pathways was not thoroughly investigated, and there was no direct validation of its effects on hepatic stellate cell activation, proliferation, or apoptosis. Consequently, the complete molecular mechanism underlying YJP’s anti-fibrotic action in the liver remains unelucidated. Furthermore, there is no clinical-level validation data to support the findings, and the synergistic mechanisms among its multiple components remain unclear. Future research should identify active components through target validation and conduct in-depth cellular experiments to elucidate the mechanism of action, thereby providing robust scientific evidence for YJP’s potential as a therapeutic agent for LF.

Yiqi Rougan Decoction (YQRG) comprises *Astragalus mongholicus Bunge (Fabaceae)*, *Atractylodes macrocephala Koidz (Asteraceae)*, *Salvia miltiorrhizae Bunge (Lamiaceae)*, *Curcuma longa L (Zingiberaceae)*, *Paeonia lactiflora Pall (Ranunculaceae)*, Cyperus rotundus L *(Cyperaceae)*, *Sargassum fusiforme Setch (Sargassaceae)*, *Trichosanthes kirilowii Maxim (Cucurbitaceae)*. Based on traditional Chinese medicine theory for treating chronic liver disease, it has demonstrated potential anti-fibrotic effects. This study investigated the intervention effects and molecular mechanisms of YQRG using CCl_4_-induced rat LF models, with colchicine as a positive control. Rats received YQRG (4.95, 9.9, 19.8 g/kg) for 5 weeks. Results indicate that YQRG significantly alleviate body weight loss and hepatic histopathological damage in model rats, reduced serum ALT and AST levels, decreased hepatic hydroxyproline and fibrosis markers HA, LN, PC-III, and IV-C deposition in liver tissue, and suppressed the expression of α-SMA. The high-dose group exhibited more pronounced effects comparable to colchicine (Xiong et al., 2021). Transcriptome analysis and validation experiments confirmed that YQRG alleviates LF by downregulating ERS, apoptosis, and autophagy-related gene expression, inhibiting the MAPK/PI3K-Akt Signaling Pathway, and simultaneously activating the AMPK/PPAR pathway to reduce ECM accumulation. This study provides multidimensional experimental evidence for YQRG’s therapeutic potential in LF, though limitations exist: The use of a single CCl_4_ induction model fails to cover metabolic and other subtypes of liver fibrosis. The synergistic mechanisms among the compound’s core active ingredients and components remain unclear, lacking component knockout or purification validation. Clinical sample data are absent, and *in vivo* experimental evidence has not been translated into *in vitro* target validation. Furthermore, the cross-regulatory mechanisms among multiple signaling pathways and the direct binding relationships between compound components and targets have yet to be elucidated. Future research should expand model types, incorporate clinical samples, and conduct *in vitro* target validation to provide more robust scientific evidence for the clinical translation of YQRG.

Additionally, we have summarized multiple TCM formulas that play a crucial role in intervening in the pathological process of LF (Table 2). These studies indicate that by targeting the regulation of the MAPK signaling pathway, these formulas can effectively inhibit the abnormal activation of HSCs, suppress the expression of local inflammatory factors in the liver, and downregulate levels of apoptosis and abnormal autophagy. This approach reduces pathological deposition of the ECM, thereby effectively reversing and alleviating LF.

**TABLE 2:** Herbal compound regulate MAPK signaling pathway to treat hepatic fibrosis.

Extract	*Origination*	Dosage	Model	Biological effects	Results	Limitations	References
THF	*Panax notoginseng (Burkill) F.H.Chen* *Coptis chinensis Franch.*	80, 160 mg/kg	CCl_4_-induced mice MCD-induced mice	ALT, AS↓ TG↓α-SMA, Collagen-I↓ TNF-α↓ IL-1β↓ CD68↓ MAPK/NF-κB↓	Improve liver function Reduce inflammatory cell infiltration Inhibit HSCs activation	The active ingredient responsible for the core effects of THF and its dose-response relationship remain unidentified	Lan et al. (2024)
FZYGHX	*Astragalus mongholicus Bunge* *Atractylodes macrocephala Koidz.* *Curcuma phaeocaulis Valeton* *Coix lacryma-jobi* var. *ma-yuen (Rom.Caill.) Stapf* *Artemisia scoparia Waldst. and Kit.* *Salvia miltiorrhiza Bunge* *Raphanus raphanistrum subsp. sativus (L.) Domin* *Ostrya virginiana (Mill.) K.Koch* *Citrus aurantium L.* *Glycyrrhiza uralensis Fisch. ex DC.*	0.45, 1.8 g/kg	CCl_4_-induced mice	ALT, AST↓ F4/80 ↓TNF-α, IL-6↓ LPS↓ LBP↓TLR4-MAPK/NF-κB↓ IL-10↑ ZO-1, Claudin-1↑Occludin↑	Reduce the liver-to-body weight ratio Ameliorate the disorder of hepatic tissue structure Reduce collagen deposition Decrease the hepatic fibrosis score	Using only the CCl_4_ model without clinical sample validation, its applicability to human liver fibrosis remains to be confirmed	Ma et al. (2025)
YJP	*Curcuma aromatica Salisb.* *Boswellia sacra Flück.* *Taraxacum mongolicum Hand.-Mazz.* *Typha angustifolia L.* *Vigna radiata (L.) R.Wilczek* *Panax notoginseng (Burk.) F.H.Chen* *Commiphora myrrha (T.Nees) Engl.*	100, 200, 300 mg/kg	CCl_4_-induced mice	ALT, AST↓ TNF-α, IL-1β↓α-SMA↓ Col1↓p-ERK, p-JNK, p-P38MAPK↓ PI3K/Akt↓	Ameliorate hepatic tissue injury Alleviate collagen deposition	The cross-regulatory relationship between the MAPK and PI3K/Akt pathways has not been thoroughly explored, and direct validation of their effects on the activation, proliferation, and apoptosis of hepatic stellate cells (HSCs) is lacking	Wu W. et al. (2019)
DHZCP	*Rheum officinale Baill.* *Scutellaria baicalensis Georgi* *Glycyrrhiza uralensis Fisch. ex DC.* *Prunus persica (L.) Batsch* *Prunus armeniaca* var. *Armeniaca* *Paeonia lactiflora Pall.* *Rehmannia glutinosa (Gaertn.) Libosch. ex DC.* *Toxicodendron vernicifluum (Stokes) F.A.Barkley* *Pometia pinnata J.R.Forst. and G.Forst.* *Terminalia chebula Retz.* *Rosa webbiana Wall. ex Royle*	50–100 μL	HSC-T6	TNF-α, IL-1β, IL-6↓ ALT, AST↓ p38 MAPK, NF-κB p65, TGF-β1↓ Smad2, Smad3↓ Smad7↑	Reduce cell apoptosis Inhibit hepatic fibrosis	No targeted validation experiments were designed, making it impossible to rule out cross-regulatory effects between this pathway and other signaling pathways	He et al. (2024)
HJRG	*Hedysarum Multijugum Maxim.* *Pueraria montana var. thomsonii Benth.* *Bupleurum falcatum L.* *Paeonia lactiflora Pall.* *Salvia miltiorrhiza Bunge* *Panax pseudoginseng Wall.* *Abrus melanospermus subsp. Melanospermus* *Phyllanthus urinaria L*	1.16, 2.32, 4.64 g/kg	CCl_4_-induced mice	TNF-α, IL-1β↓ IL-6↓ Cox2↓ iNOS↓ MDA↓ MPO↓ α-SMA↓ desmin↓ vimentin↓ HA, LN, PCIII, ColIV↓ SOD↑ GSH↑	Alleviate hepatic inflammatory response Block oxidative stress Ameliorate hepatic tissue injury	Without validating the actual bioavailable components using *in vivo* pharmacokinetic data, key bioactive substances may be overlooked	Cai et al. (2022)
ZQD	*Arnebia euchroma (Royle ex Benth.) I.M.Johnst* *Paeonia suffruticosa Andrews* *Paeonia lactiflora Pall* *Rehmannia glutinosa (Gaertn.) Libosch. ex DC.* *Panax pseudoginseng Wall* *Atractylodes lancea (Thunb.) DC.* *Phellodendron amurense Rupr.* *Sophora flavescens Aiton* *Sedum sarmentosum Bunge*	0.5, 1, 1.5 mL/100 g	CCl_4_-induced mice	α-SMA↓TNF-α, IL-6↓NF-κB↓ MAPK↓NF-κB p65↓ERK, JNK, p38↓	Inhibit HSCs activation Improve liver function Alleviate hepatic inflammatory response Ameliorate hepatic tissue structural disorder	Animal studies have relatively short durations and lack long-term safety and efficacy data	Zhou et al. (2021)
		2.75, 5.5, 11 g/mL	LX-2 cells				
YQRG	*Astragalus mongholicus Bunge.* *Atractylodes Macrocephala Koidz.* *Salvia miltiorrhizae Bunge* *Curcuma longa L.* *Paeonia lactiflora Pall.* *Cyperus rotundus L.* *Trichosanthes Kirilowii Maxim.*	4.95, 9.9, 19.8 g/kg	CCl_4_-induced rats	ALT, AST↓HA, LN, PC-III, IV-C↓ α-SMA↓MAPK/PI3K-Akt ↓ AMPK/PPAR↑	Ameliorate hepatic tissue injury Inhibit HSCs activation Improve liver function	Other types of liver fibrosis models, such as metabolic fibrosis, were not included; the core active components and synergistic mechanisms within the compound formulation remain unclear, and there is a lack of component knockout or purification validation	Xiong et al. (2021)

## 7 Discussion

LF represents a critical pathological stage in the progression of various chronic liver diseases toward cirrhosis and hepatocellular carcinoma. Its core pathological features include abnormal activation of HSCs, pathological deposition of ECM, and imbalance in the hepatic inflammatory microenvironment. Without timely intervention, this irreversibly drives disease progression to end-stage liver disease, posing a severe threat to global public health. Although its pathological mechanisms remain incompletely elucidated, abnormal signaling pathway regulation is recognized as a key driver of disease progression. Among these, sustained activation of MAPK has been confirmed as a core driving event. Consequently, targeting MAPK has emerged as a critical direction for precision therapy in LF. Natural drugs and natural formulas, leveraging their unique advantages of extensive availability, structural diversity, relatively low toxicity, and multi-target synergistic regulation, have garnered significant attention in the field of LF treatment. Natural bioactive components such as flavonoids, terpenoids, and alkaloids, along with TCM formulas, can synergistically exert anti-inflammatory, antioxidant, anti-HSCs activation, and ECM metabolic balance effects by regulating key nodes in MAPK. This effectively delays or even reverses the progression of LF, demonstrating greater therapeutic potential compared to traditional single-target therapeutic drugs.

Although the findings summarized in this review confirm the significant potential of natural drugs targeting the MAPK pathway for treating LF, numerous core issues in this field remain to be addressed, pointing to clear directions for future research. First, the precision and systematic nature of the mechanisms of action require in-depth analysis. Existing studies primarily confirm the regulatory effects of natural products on the phosphorylation levels of key MAPK pathway subtypes. However, the direct binding sites and interaction patterns between core active components and MAPK pathway molecules remain unclear. Furthermore, the cross-regulatory networks between the MAPK pathway and other pro-fibrotic pathways have not been thoroughly analyzed, particularly lacking systematic research on the multi-component combination regulation of pathway networks in TCM formulas. Future mechanistic studies should leverage advanced technologies such as molecular docking, high-resolution proteomics, single-cell sequencing, and CRISPR-Cas9 gene editing to identify direct binding targets between natural active components and key MAPK pathway molecules, thereby elucidating their structure-activity relationships. Simultaneously, for TCM formulas, integrating network pharmacology, component knockout, and combination validation methods should elucidate the synergistic regulatory mechanisms of multi-component combinations on the MAPK pathway and its cross-talk pathways. This will identify core bioactive components and synergistic combinations, thereby refining the molecular regulatory theory of natural products targeting MAPK pathway. Second, the severe shortage of integrated pharmacokinetic-pharmacodynamic analysis and clinical translation support data constitutes a core bottleneck constraining the clinical application of natural products. Existing research predominantly relies on animal models such as chemically induced fibrosis and bile duct ligation, which struggle to replicate the etiological heterogeneity and complex pathological microenvironment of clinical LF. Furthermore, most natural compounds lack systematic pharmacokinetic studies, leaving their oral bioavailability, metabolic profiles, optimal dosages, and administration routes undefined. Large-scale, long-term, multicenter clinical trials to validate their efficacy and safety are also absent. Future efforts should prioritize establishing clinically relevant, etiology-specific LF models and conducting systematic pharmacokinetic-pharmacodynamic linkage studies to elucidate the correlation between absorption, distribution, metabolism, and excretion characteristics of core active ingredients and their antifibrotic efficacy. For natural compounds with poor oral absorption and rapid metabolism, develop novel delivery systems such as liver-targeted nanocarriers and liposomes to enhance bioavailability and hepatic targeting. Concurrently, design randomized, controlled, blinded multicenter clinical trials. Integrate LF etiological classification and specific biomarker detection to validate the efficacy and safety of natural products in patients with different stages of LF and special populations, providing robust evidence for clinical translation. Third, existing research designs exhibit numerous shortcomings, and an evaluation system for synergistic components in TCM formulas remains unestablished. Some studies lack clear dose-response analysis, employ limited positive controls, and omit long-term toxicity assessments, compromising result reliability. TCM formula research often focuses on overall efficacy while neglecting screening and validation of core active components, leaving the scientific rationale for ingredient combinations unclear. Future efforts should optimize study designs by incorporating multi-tiered dose groups and diversified positive controls, alongside long-term toxicology studies to define safe dosage ranges and potential adverse reactions for natural products. For TCM formulas, chemical isolation, pharmacological validation, and multi-omics analysis should be integrated to screen and validate core bioactive components and their optimal combination ratios, elucidating synergistic mechanisms among different components in targeting the MAPK pathway. Concurrently, multi-omics technologies should be employed to identify prognostic and therapeutic biomarkers for LF, enabling the development of personalized intervention strategies tailored to distinct etiologies and disease stages. Exploring the potential synergistic application of natural products with first-line anti-fibrotic drugs could achieve enhanced therapeutic efficacy with reduced toxicity.

In summary, this review systematically reviews the latest research advances on natural compounds and TCM formulas regulating the MAPK pathway to intervene in LF, clarifying the unique advantages and core value of this research direction. Although current research still faces challenges such as insufficient mechanism elucidation, inadequate clinical translation support, and imperfect study designs, anti-LF research targeting the MAPK pathway with natural products remains a highly promising innovative direction. Future efforts should integrate molecular biology, TCM pharmacology, pharmacokinetics/pharmacodynamics, and clinical medicine through multidisciplinary collaboration. Breakthroughs in precise mechanism elucidation, pharmacokinetic-pharmacodynamic integration, development of clinically relevant models, and optimized study designs are anticipated. This approach holds promise for developing safe, effective, and highly targeted natural product-based anti-LF drugs or treatment regimens, advancing the therapeutic landscape for LF. It could propel LF therapy from “symptomatic relief” to “precision reversal,” offering new strategies and options for the clinical management of chronic liver disease and LF.

## Glossary

LF: Liver Fibrosis
HSC: Hepatic Stellate Cell
MAPK: Mitogen-Activated Protein Kinase
ERK: Signal-Regulated Kinase
JNK: c-Jun N-Terminal Kinase
TCM: Traditional Chinese Medicine
WHO: World Health Organization
ECM: Extracellular Matrix
OS: Oxidative Stress
c-Fos: Cellular Proto-Oncogene Fos
Elk-1: ETS Domain-Containing Protein Elk-1
α-SMA: α-Smooth Muscle Actin
MKK: MAP Kinase Kinase
TNF-α: Tumor Necrosis Factor-α
IL-1β: Interleukin-1β
PDGF: Platelet-Derived Growth Factor
ATF2: Activating Transcription Factor 2
MEF2: Myocyte Enhancer Factor 2
MK2: MAPK-Activated Protein Kinase 2
MMP: Matrix Metalloproteinase
IL-6: Interleukin-6
TGF-β: Transforming Growth Factor-β
VEGF: Vascular Endothelial Growth Factor
ROS: Reactive Oxygen Species
SOD: Superoxide Dismutase
Nrf2: Nuclear Factor Erythroid 2-Related Factor 2
TBIL: Total Bilirubin
ALT: Alanine Aminotransferase
AST: Aspartate Aminotransferase
HA: Hyaluronic Acid
LN: Laminin
IV-C: Type IV Collagen
PIIIP: Procollagen III Peptide
IκBα: Inhibitor of Nuclear Factor κB α
NF-κB: Nuclear Factor κB
Bcl-2: B-Cell Lymphoma 2
Bax: Bcl-2-Associated X Protein
Cox-2: Cyclooxygenase-2
TIMP-1: Tissue Inhibitor of Metal Protease 1
OPC: Oligomeric Proanthocyanidins
LPS: Lipopolysaccharide
PI3K: Phosphatidylinositol 3-Kinase
Akt: Protein Kinase B
IH: Isorhamnetin
BDL: Bile Duct Ligation
**CCl**_**4**_: Carbon Tetrachloride
LC3: Microtubule-Associated Protein 1 Light Chain 3
PPAR-γ: Peroxisome Proliferator-Activated Receptor-γ
BDL: Bile Duct Ligation
TAK: Transforming Growth Factor-β Activated Kinase 1
FoxO3a: Forkhead box O3a
MASH: Metabolic Dysfunction–Associated Steatohepatitis
KA: Kaempferol
GTIN: Gossypetin
GSP: Grape Seed Proanthocyanidin
APG: Apigenin
DIO: Dioscin
AS-IV: Astragaloside IV
HSYA: Hydroxysafflor Yellow A
4-MSP: 4-Methoxy Sulfonyl Paeonol
DHZ: Dehydrozingerone
CGA: Chlorogenic acid
BA: Betulinic acid
Tan IIA: Tanshinone IIA
MGIVE: Mogroside IVE
SCA: Schisantherin A
OJP-W2: Ophiopogonis Radix Inulin New Series Fructan
Sal B: Salvianolic acid B
TPLR: Total polyphenolic glycoside extract
LH: Lamiophlomis Herba
GBE: Ginkgo biloba extract
CGEA: Cichorium pumilum Jacq ethyl acetate extract
SE: Sasa extract
EPP: Pomegranate peel extract
THF: Tianhuang Formula
FZYGHX: Fuzheng Yanggan Huaxian Decoction
YJP: Yu Jin Pulvis
DHZCP: Da Huang Yu Chong Wan
ZQD: Zi Qi Decoction
HJRG: Huangjia Ruangan Granule
YQRG: Yiqi Rougan Decoction
